# Carbon Fiber Oxidation in 4D

**DOI:** 10.1002/adma.202502007

**Published:** 2025-07-14

**Authors:** Benjamin M. Ringel, Federico Semeraro, Joseph C. Ferguson, Harold S. Barnard, Bruno Dias, Christian M. Schlepütz, Edward S. Barnard, Sam Schickler, Kara Levy, Shawn Shacterman, Talia Benioff‐White, Julian Davis, Alastair A. MacDowell, Dilworth Y. Parkinson, Francesco Panerai

**Affiliations:** ^1^ Department of Aerospace Engineering, Grainger College of Engineering University of Illinois at Urbana‐Champaign 104 S Wright Street Urbana IL 61802 USA; ^2^ Analytical Mechanics Associates NASA Ames Research Center Moffett Field CA 94035 USA; ^3^ Department of Mechanical Engineering Stanford University 440 Escondido Mall Stanford CA 94305 USA; ^4^ Advanced Light Source Lawrence Berkeley National Laboratory Berkeley CA 94720 USA; ^5^ Swiss Light Source Paul Scherrer Institute Forschungsstrasse 111 5232 Villigen Switzerland; ^6^ Molecular Foundry Lawrence Berkeley National Laboratory Berkeley CA 94720 USA

**Keywords:** ablation, carbon fiber, carbon oxidation, thermal protection system, x‐ray microtomography

## Abstract

The oxidation of carbon fibers at high temperatures is the primary degradation process in the thermal protection system of many hypersonic flight vehicles. Predicting the rate and the extent of oxidation is critical to ensure a safe and effective design. An oversized thermal protection system adds unnecessary mass, while an under‐designed one risks system failure and mission loss. Resolving high‐temperature material degradation due to oxidation has been a long‐standing challenge in designing for re‐entry flight environments. Using time‐resolved in situ X‐ray microtomography, the oxidation of carbon fibers at high temperatures is directly imaged, resolving the two limiting degradation regimes: diffusion‐ and reaction‐limited. The ability to resolve material degradation in time at the sub‐micron scale sheds light on the ablation phenomenon and enables predictions of material constitutive properties evolving in time, with profound implications on the ability to model the aerothermal response of heat shield materials in hostile environments.

## Graphical abstract

The oxidation of porous carbon fiber heat shield material at high temperatures is imaged using time‐resolved synchrotron X‐ray microtomography. Resolving the interplay between species diffusion and carbon‐oxygen reaction rate reveals spatially and temporally varying degradation across oxidation regimes, with profound implications for in‐depth material properties, structural integrity, and the predictive design of aerospace thermal protection systems.

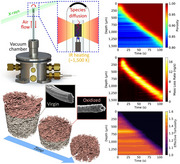

## 1 Introduction

Hypersonic vehicles piercing through the atmosphere at extreme velocities endure harsh aerothermal loads as their kinetic energy is rapidly converted to thermal energy across shock waves. It is the job of the thermal protection system (TPS) to shield the vehicle, a task met with advanced thermo‐structural materials. For the fastest flight trajectories, such as those flown during atmospheric (re‐)entry, ablative composites are adopted, as they are capable of dissipating extreme aerothermal loads through sacrificial decomposition. Carbon fibers are a preferred material choice in ablative heat shields, owing to their ability to retain thermomechanical properties at temperature and the capacity to engineer desirable anisotropic architectures by controlling fiber orientation. Carbon ablation in composite heat shield materials is an engineering challenge with strong relevance to fields such as fire safety, combustion, biomass conversion, and rocket science.^[^ ^1^, ^2^, ^3^, ^4^ ^]^

X‐ray micro‐computed tomography (μ‐CT) is an effective tool to analyze anisotropic fibrous structures. It allows for 3D resolution at scales below a micrometer and provides a Cartesian base for computational material simulations.^[^ ^5^, ^6^, ^7^, ^8^, ^9^, ^10^ ^]^ Moreover, advances in high‐temperature test environments deployed in situ at synchrotron μ‐CT beamlines have brought unique insight into material evolution and phenomena at extreme conditions.^[^ ^11^, ^12^, ^13^, ^14^, ^15^ ^]^ The emergence of high temporal resolution capabilities^[^ ^16^ ^]^ is an attractive avenue for ablative materials undergoing rapid high‐temperature degradation.

The ablation of carbon TPS materials involves multiple mass‐removal processes,^[^ ^3^ ^]^ among which oxidation is primary, leading to surface recession and changes in aerodynamic shape. High‐temperature oxidation of carbon‐based materials stands on a large body of literature,^[^ ^17^ ^]^ yet models fail to accurately predict across all flight regimes, and effective thermal and mass transport properties remain poorly defined for a decomposing material. Molecular beam and high‐enthalpy wind tunnel experiments^[^ ^18^, ^19^, ^20^, ^21^, ^22^, ^23^, ^24^ ^]^ have enabled the development of high‐fidelity kinetic models for carbon ablation in air,^[^ ^25^, ^26^, ^27^ ^]^ for which validation across regimes is yet to be accomplished. To this end, experimental data that cover the full spectrum of oxidation regimes are still sparse. The theory of porous ablators predicts the emergence of limiting regimes for high‐temperature oxidation,^[^ ^28^, ^29^, ^30^, ^31^ ^]^ described by the ratio of reaction to diffusion rates, also known as Thiele number, Φ. A high Thiele number (Φ ≫ 1) corresponds to fast reactions with slow diffusion, hence oxidation is diffusion‐limited. Conversely, a low Thiele number (Φ ≪ 1) reflects slow reactions relative to diffusion, resulting in reaction‐limited oxidation. For intermediate values (Φ ≈ 1), diffusion and reactions occur on comparable timescales, defining the mixed oxidation regime. In this work, we present experiments that resolve the limiting Thiele regimes for a low‐density carbon fiber material interacting with air, demonstrating the ability to quantify degradation processes with microscale and time resolution, that is in 4D, and introducing a strategy to resolve effective material properties as a function of decomposition.

## 2 Results and Discussion

### 2.1 Real‐Time μ‐CT of High‐Temperature Oxidation

**Figure 1:**
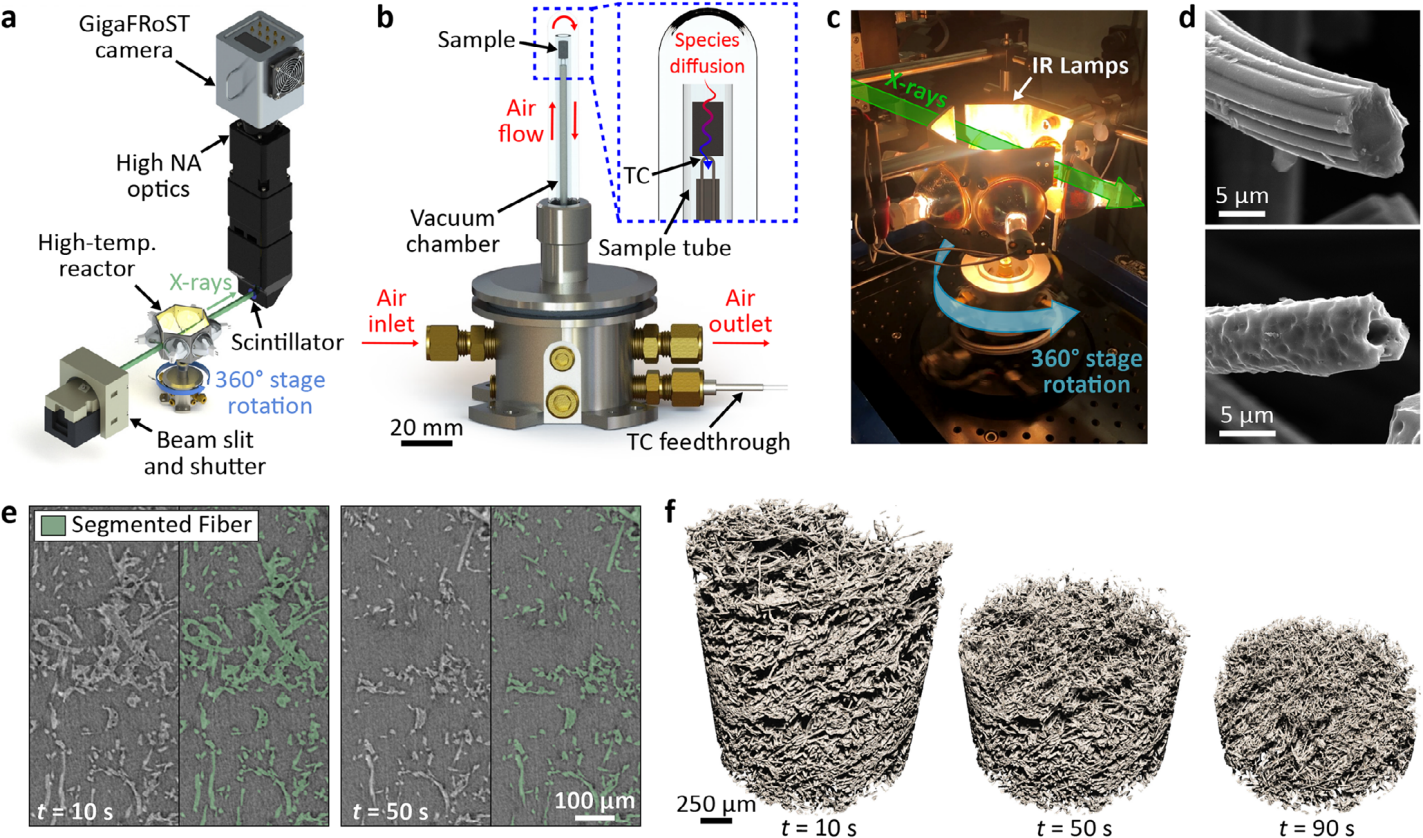
In situ μ‐CT imaging of high‐temperature carbon fiber oxidation in air. a) Schematic of the reactor and collection optics arrangement in the beamline X‐ray path. b,c) Schematic and photograph of the high temperature operation of the reactor. d) Pre‐ and post‐mortem scanning electron micrographs of the sample material showing the characteristic crenulated cross‐section of a virgin rayon‐based carbon fiber; the pitted fiber surface is a signature of oxidized material.^[^ ^32^ ^]^ Notice also the reduction in fiber diameter due to oxidation and the opening of internal fiber lumen. e) Reconstructed slices for virgin and oxidized material with overlaid segmentation. f) Volume‐rendered 4D μ‐CT of sample oxidation.

A custom‐built reactor was developed to operate in situ at the TOMCAT beamline of the Swiss Light Source (SLS) to perform real‐time X‐ray μ‐CT imaging of a carbon fiber material subjected to oxidation at temperature. Heating was achieved using six 150 W halogen lamps, focused on a micro‐plug sample of the porous material, suspended in a controlled atmosphere quartz chamber (**Figure** **1**b). Nine experiments were performed at controlled environmental pressures between 2.7 and 101.3 kPa and temperatures between 945 and 1445 K, as measured by a thermocouple probe in contact with the bottom surface of the micro‐plug. Test conditions were optimized based on experimental constraints and estimated Thiele regimes along reference flight trajectories for porous carbon ablators. With the sample interference fit into a quartz capillary tube and air flow supplied to the top of the sample, a diffusion flux of reactants was established toward and through the porous material domain (Figure 1c). As the sample oxidized, synchrotron X‐rays (Figure 1a,c) were used to collect tomography scans with an effective voxel size of 0.81 μm/voxel at a temporal resolution of one full tomographic scan per second, allowing 4D imaging of the degradation phenomenon at various temperatures and pressures. Further details on the experimental setup and the selection of test conditions are provided in the Supporting Information.

The micrographs of virgin and oxidized fibers, shown in Figure 1d, highlight details of the degradation processes. The crenulated profile of the virgin fiber is shrunk into a smaller diameter cross‐section, nearly circular in shape. Clear signs of pitting are visible over the fiber surface. Progressive degradation also leads to the opening of the hollow fiber core (lumen). Both pitting and lumen opening contribute to local changes of available reactive surface area for oxidation to occur.^[^ ^32^, ^33^, ^34^ ^]^ The scale (≲2 μm) of these features is below the spatial resolution of the present 4D imaging.

Convolutional neural networks (CNNs) (Figure 1e) were used to correct for void phase noise, motion artifacts, and low contrast between fibers and pores in reconstructed datasets,^[^ ^35^ ^]^ resulting in high‐quality segmentation of large tomographic sequences. For the nine tests discussed in this work (*cf*. Table S1, Supporting Information), a custom 2D U‐Net CNN was trained and applied to over 1200 tomographies, each of 2016 × 2016 × 2016 voxels. The resulting segmented datasets were cropped to the sample field of view and used to quantify material evolution while undergoing oxidation (Figure 1f).

### 2.2 Resolution of Limiting Oxidation Regimes

**Figure 2:**
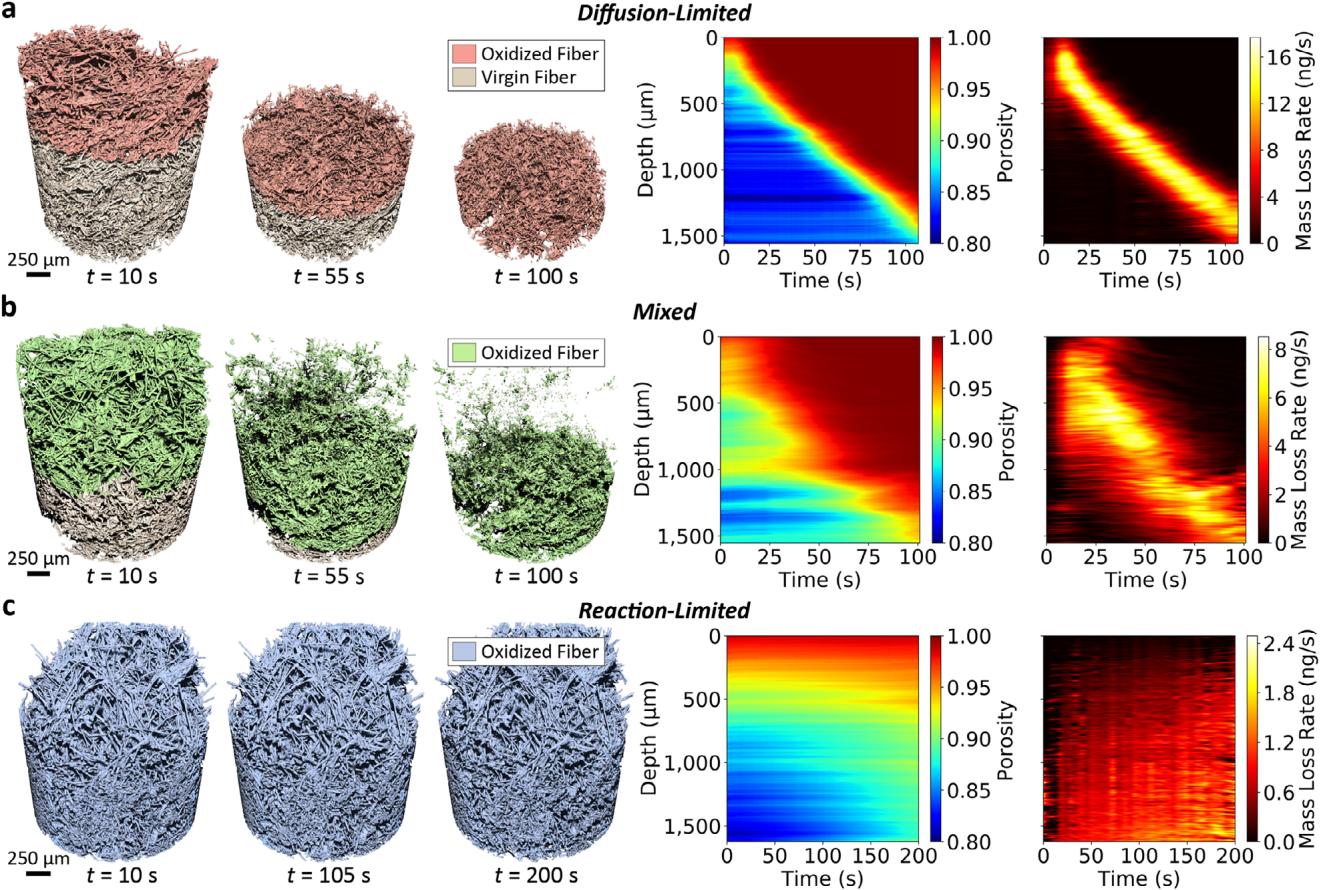
Resolution of carbon fiber oxidation regimes. From left to right: Volume rendered μ‐CT of progressive carbon fiber oxidation at three discrete time instances (left), with colored regions representing the oxidized volume; space‐time contour maps of porosity (center) and mass loss rate (right) evolution. Spatial information in the contour map is displayed as slice‐wise in‐depth averages (*cf*. Section S1.6, Supporting Information). a) Diffusion‐limited regime (test 1 – 1320 K, 100 kPa), with prominent degradation confined in a narrow region close to the surface. b) Mixed regime (test 2 – 1060 K, 100 kPa), with significant degradation occurring both near the surface and in‐depth. c) Reaction‐limited regime (test 3 – 945 K, 100 kPa), with mild degradation across the entire available volume.

Time‐resolved regimes of carbon fiber oxidation are presented in **Figure** **2** as sequences of 3D‐rendered μ‐CT data. Porosity and carbon mass loss rate contour maps show the evolution of such properties in time and depth within the porous medium. The corresponding videos are provided in Movie S1 (Supporting Information), for the diffusion‐limited (test 1), mixed (test 2), and reaction‐limited (test 3) cases, respectively.

At the highest temperature, diffusion‐limited oxidation (Figure 2a) features aggressive oxidation near the sample surface, with no significant degradation occurring in depth. In this regime, material reactivity exceeds diffusive transport, leading to prevalent oxygen consumption in a narrow region near the receding surface. Both porosity and rate of carbon loss show a steep gradient, limited to a narrow zone of <500 μm in depth.

Mixed‐regime oxidation is observed at intermediate temperatures. At these conditions, mass transport and reaction kinetics compete with comparable rates, leading to strong degradation at the surface and progressively weaker oxidation in depth (Figure 2b). The evolutions of porosity and mass loss over time feature spatially wider gradients, far larger than those observed in the diffusion‐limited case. Over half of the receding volume is affected by in‐depth oxidation. This substantial increase in affected depth has profound implications for ablation response during hypersonic flight. Deeper degradation weakens mechanical properties through the thickness, which may result in augmented erosion (up to the millimeter scale) when the material is exposed to the high‐shear stress flow.

At the lowest temperatures, in the reaction‐limited regime, oxidation is hardly perceivable (Figure 2c). Weak and nearly uniform degradation throughout the volume is the manifestation of diffusion processes becoming faster than the interaction of oxygen with the carbon fibers. Mass loss rate is on the order of ≈1 ng s^−1^, twentyfold lower than that measured for the diffusion‐limited case. While 3D visualizations show little change in sample geometry over time, a gradual increase in sample porosity throughout the domain as the experiment progresses demonstrates fully volumetric oxidation. Notably, we found this slow and seemingly benign degradation process can lead to structural failure when fiber and interface bonding are sufficiently oxidized. In prior work, microstructural weakening of the carbonized binder that links the fibers has been attributed to its higher reactivity relative to the fibers themselves, potentially increasing its susceptibility to oxidative erosion.^[^ ^33^ ^]^ This preferential degradation compromises the integrity of fiber‐fiber connections, mechanically weakening the composite despite the absence of surface recession. Movie S2 (Supporting Information) (test 6) is a case in point, showing the abrupt collapse of the fiber structure under reaction‐limited conditions.

### 2.3 Quantitative Oxidation Analysis

**Figure 3:**
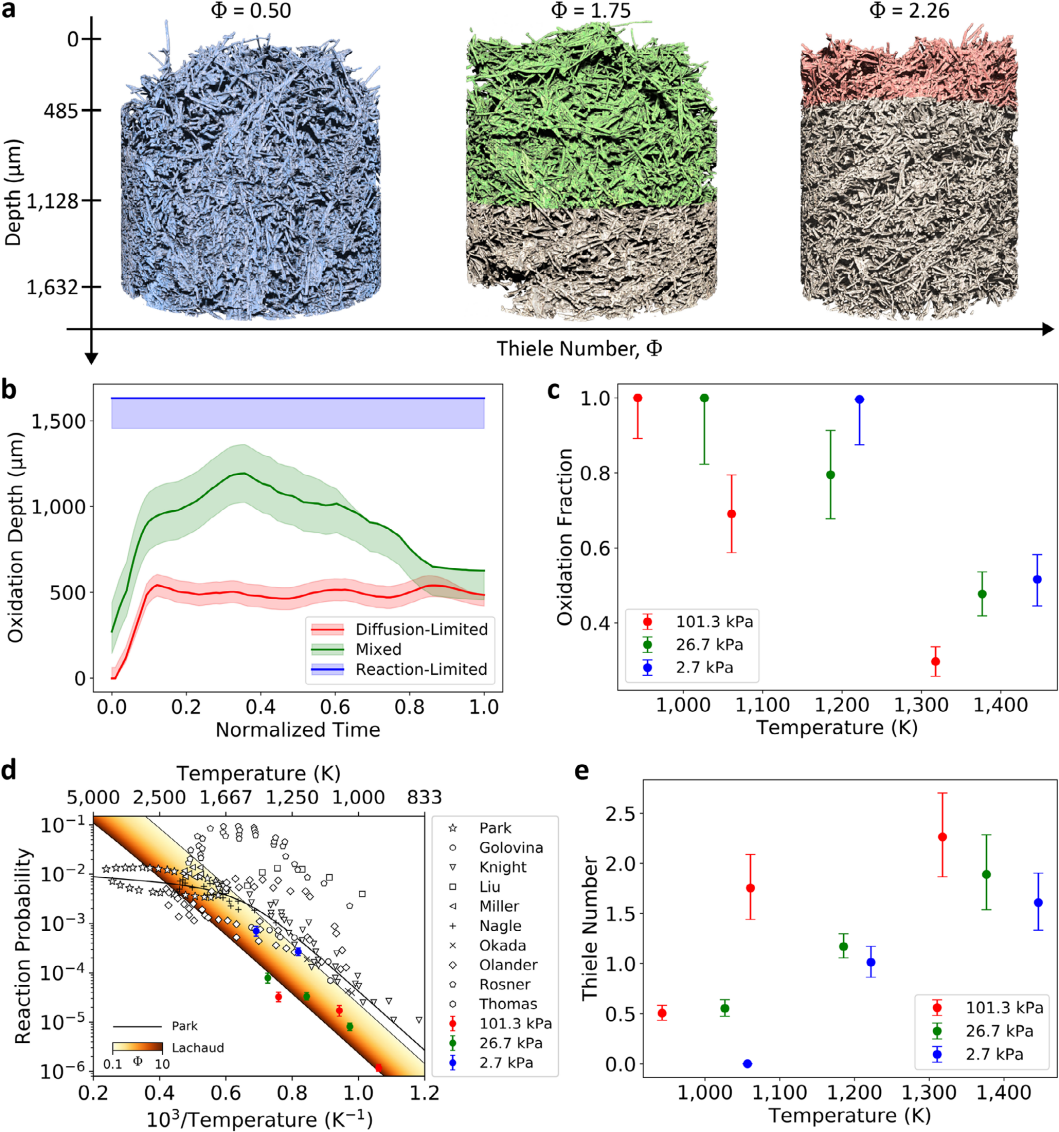
Quantification of oxidation. a) Comparative 3D visualization of measured steady‐state oxidation depth for experiments at atmospheric pressure. b Oxidation depth over time, demonstrating a brief transient period followed by steady‐state oxidation, where diffusion and reaction processes are at equilibrium. c) Oxidation fraction, the ratio between steady‐state oxidation depth and the full depth of the sample, for experiments at all pressure conditions. Test 9 has not been included, as no oxidation was measured during this experiment (see the Supporting Information for more details). d) Calculated reaction probability of diatomic oxygen with solid carbon from 4D μ‐CT data (shown in color) compared to previous studies on graphite^[^ ^17^, ^36^, ^37^, ^38^, ^39^, ^40^, ^41^, ^42^, ^43^, ^44^ ^]^ and oxidation models from literature.^[^ ^17^, ^31^ ^]^ Lachaud's model uses Equation (4) and effective reactive surface area correction in Equation (27) from the respective reference.^[^ ^31^ ^]^ e) Computed Thiele number for all nine experiments, demonstrating temperature and pressure effects on diffusion‐reaction competition within the porous material.

The oxidation depth is defined as the distance from the sample surface to the deepest location exhibiting measurable oxidation (see the Supporting Information for details). This quantity was tracked over time for all nine experiments, with steady‐state values visualized as the shaded regions in **Figure** **3**a. Time‐resolved oxidation depth measurements are shown in Figure 3b for tests 1, 2, and 3. The diffusion‐limited and mixed regimes feature a short initial transient, where the oxidation depth grows as the sample begins to recess, and a subsequent region where diffusion and reaction processes reach equilibrium. In the diffusion‐limited case (test 1), the oxidation depth stabilizes to a steady state value of approximately 485 μm, which remains constant throughout the experiment. The mixed regime case (test 2) shows a prominent increase in measured depth to an average value of 1128 μm and gradual decay in the late phase of the experiment. This decay is attributed to the oxidation zone extending beyond the imaging field of view as oxidation progressed, with additional contributions by large local porosities observed in this specific sample (these can be seen in Figure 2a – the mixed regime case – at depth near 1000 μm, as well as at the bottom of the domain). In the reaction‐limited case, oxidation was measured to extend throughout the whole domain (1632 μm) for the entire duration of the experiment.

Figure 3c compares oxidation fractions (i.e., the volume fraction of the sample domain subjected to oxidation) for all the experiments, with the highest values found for the rate‐limited cases. The observed decrease in oxidation fraction with increasing temperature is evidence of conditions that trend deeper into transport‐limited regimes. This observation is consistent with previous findings from experiments in an inductively coupled plasma wind tunnel, where evidence of pure surface ablation was found for carbon fibers at surface temperatures exceeding 2000 K.^[^ ^23^ ^]^ A similar observation was reported in the theoretical work by Ferguson et al., where oxygen penetration was predicted to be approximately 150 μm ^[^ ^28^ ^]^ at surface temperatures of 2395 K, in agreement with arcjet experiments. The effects of pressure are also demonstrated in Figure 3c, with oxidation fraction increasing as pressure decreases for experiments at similar temperatures. This is attributed to the lower pressure promoting higher diffusivity, in turn allowing for deeper oxygen penetration into the porous medium, a result theoretically shown by the modeling work of Lachaud et al.^[^ ^31^ ^]^ Note that the nondimensional Thiele analysis seen in Figure 3e is consistent with the quantified oxidation fractions, with higher Thiele numbers (Φ ≳ 1.5) obtained for the diffusion‐limited cases and lower Thiele numbers (Φ ≲ 1.5) for the reaction‐limited ones. Thiele values also agree well with those reported for past flow tube oxidation experiments on FiberForm.^[^ ^32^ ^]^

Reaction probability – defined as the ratio of carbon removal to incident oxygen flux – is calculated from measured sample mass loss, oxidized surface area, and experimental conditions. Our results trend well with previous studies on carbon‐oxygen reactions in graphite,^[^ ^17^, ^36^, ^37^, ^38^, ^39^, ^40^, ^41^, ^42^, ^43^, ^44^ ^]^ though deviations are observed at higher pressures. These discrepancies are in part attributed to the specific carbon material investigated herein, versus reported data for graphite. At low pressure, computed reaction probabilities align with previous measurements, with an average percent error of 54% relative to Park's 1976 model.^[^ ^17^ ^]^ In contrast, tests at 26.7 and 101.3 kPa exhibit larger deviations, with average errors of 90% and 91%, respectively. This trend is consistent with the role of diffusivity in porous media. At low pressures, rapid oxygen transport enables effective reactivity to approach the intrinsic fiber reactivity, whereas at higher pressure, slower diffusion limits the overall rate.^[^ ^31^ ^]^ Importantly, unlike prior studies, we account for reactive surface area in the quantification of reactivity (*cf*. Supporting Information for details), which is directly measured from the μ‐CT data, resulting in lower estimated values compared to approximations based on sample geometry. Good agreement is found when comparing with Lachaud's reactivity model for O_2_, which accounts for regime‐dependent effective reactive surface area.^[^ ^31^ ^]^

### 2.4 Degradation‐Resolved Material Properties

**Figure 4:**
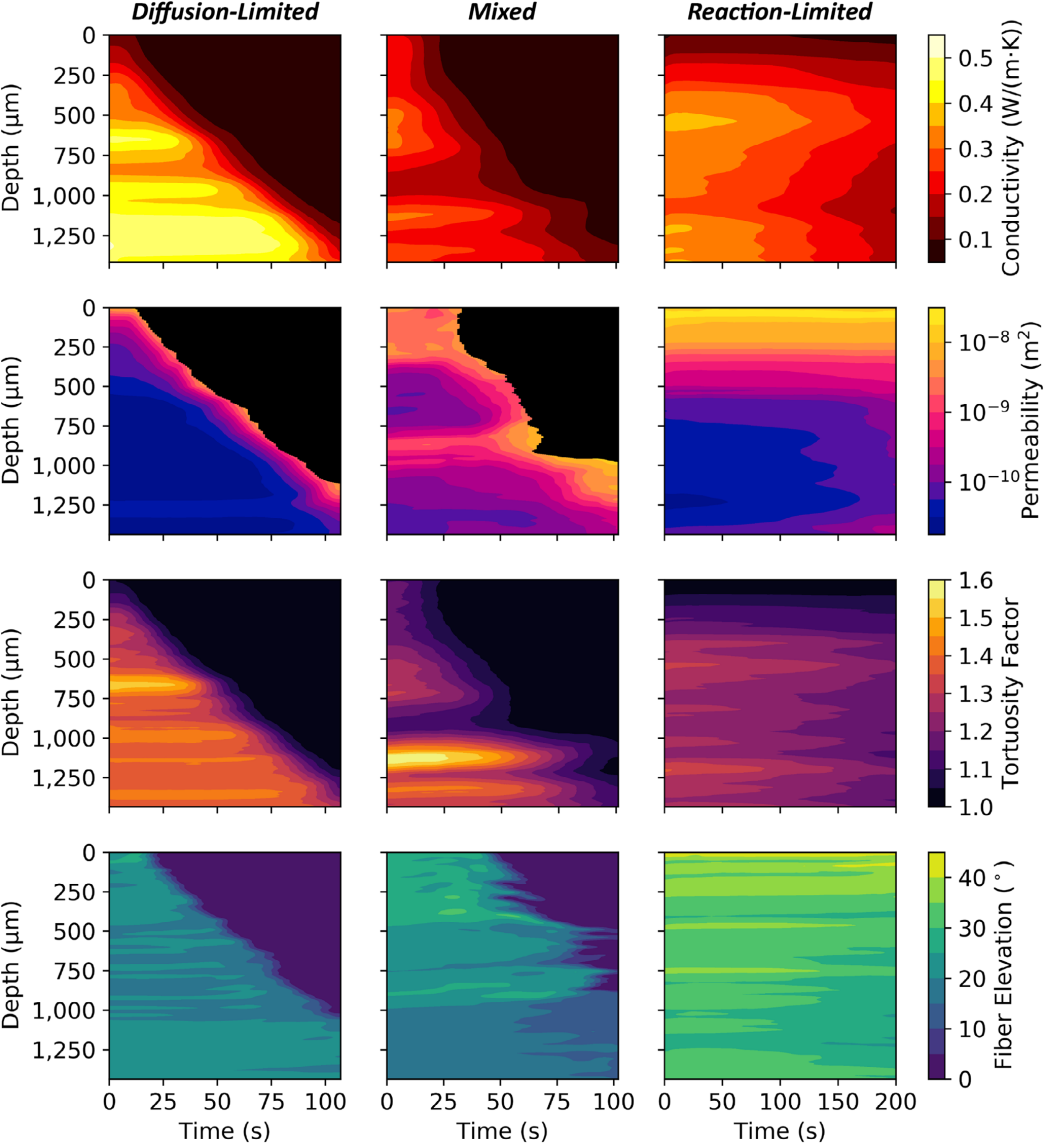
Computation of material property evolution as a function of depth and time. Effective thermal conductivity, effective permeability, effective tortuosity factor, and fiber elevation for diffusion‐limited (test 1 – 1320 K, 100 kPa), mixed (test 2 – 1060 K, 100 kPa), and reaction‐limited (test 3 – 945 K, 100 kPa) experiments. Results demonstrate the effect of oxidation on TPS material performance, with diffusion‐limited regimes yielding strong gradients in material properties near the sample surface and reaction‐limited regimes exhibiting gradual change in material throughout the sample domain in time. Free‐fluid regions of the sample – where porosity approaches unity – are shown in black for visualizations of effective permeability.

Time‐resolved μ‐CT scans enabled computations of effective material properties as a function of degradation. This was achieved by performing over 1600 supercomputing simulations using NASA's Porous Microstructure Analysis (PuMA) software.^[^ ^6^, ^7^, ^8^, ^9^, ^10^, ^45^ ^]^ The results are summarized in **Figure** **4**, where through‐thickness thermal conductivity, permeability, tortuosity factor, and fiber elevation are shown for the three regimes (tests 1–3). The evolution of each effective property is illustrated as an intensity contour map over space (slice‐wise average in‐depth) and time (*cf*. Supporting Information for further details on calculations and averaging).

A few key patterns emerge from this analysis. Overall, the porous material becomes a poorer conductor and filter during degradation due to an increase in void fraction. This is evident from the decreasing effective thermal conductivity, increasing permeability (ease of momentum transport), and decreasing tortuosity (opposition to diffusive transport) as oxidation progresses. Changes in effective properties correlate with the different porosity evolutions observed for the different oxidation regimes. In the diffusion‐limited case, as porosity changes locally close to the surface, effective properties feature steeper gradients in space, transitioning from virgin bulk values to values in air over the narrow degradation zone. Conversely, in the reaction‐limited case, as porosity changes uniformly across the entire material volume, effective properties primarily demonstrate gradients in time. These gradients are generally milder than those of the diffusion‐limited case due to a slower degradation of the material.

The absolute value of effective properties is primarily affected by the local density of the material. Variability is driven by the inherent heterogeneity of FiberForm and the sample preparation method (*cf*. Experimental Section) leading to variability in effective properties. For example, results for effective thermal conductivity show variations in virgin values across experiments, with the diffusion‐limited case (test 1) having a conductivity of 0.35–0.45 W(m·K)^−1^ versus 0.2–0.35 W(m·K)^−1^ for the mixed regime (test 2) and reaction‐limited (test 3) cases. This discrepancy is attributed to the different initial porosity of each sample, with test 1 featuring a higher fiber fraction compared to tests 2 and 3 (also seen qualitatively in the volume renderings of Figure 2). The tight correlations between effective properties and porosity are also noticed in the horizontal banding of each property. Because the material has a layered fiber density, with preferential compaction in strata of 150 to 300 μm, the effective properties show low/high variations through the thickness of the material. Overall, the range of porosity we observe captures the true heterogeneity of FiberForm and its effective properties. This stochastic nature is a key feature of carbon‐bonded carbon fiber preforms currently in use that could be mitigated by improvements to processing and sample preparation. It must be accounted for in material selection and robust calculations of design margins.

Values of the effective thermal conductivity predicted in this study are slightly higher than those reported in previous computations for the same material;^[^ ^9^, ^29^ ^]^ the difference arises because the previous work^[^ ^46^ ^]^ used room temperature values for the solid‐phase thermal conductivity, whereas the present calculations utilize values corresponding to the measured temperature. Permeability shows similar trends as the effective thermal conductivity. Virgin scans with nominal porosity have average values between 2.5E‐11 m^2^ and 1.8E‐10 m^2^ (with higher values relegated to the mixed regime case), in excellent agreement with both numerical predictions using Direct Simulation Monte Carlo (DSMC)^[^ ^47^ ^]^ and reported experimental measurements for the material as a function of temperature and pressure.^[^ ^48^ ^]^ Effective tortuosity shows in‐depth variations and distinct behavior between experiments, which, like for permeability and conductivity, correlate to material porosity. Bulk virgin tortuosity at the beginning of each experiment was found to be approximately 1.36, 1.26, and 1.23 for the diffusion‐limited, mixed, and reaction‐limited cases, respectively. Previous experimental and computational studies have shown that virgin FiberForm has a bulk tortuosity of approximately 1.23–1.25,^[^ ^29^, ^49^ ^]^ showing excellent agreement with the mixed and reaction‐limited cases.

Calculations of fiber elevation highlight the layered structure of the material, contributing to the property in‐depth variation (see above). Fibers near receding surfaces in the diffusion‐limited and mixed regime cases exhibit elevation change before being fully ablated, indicating the gasification process contributing to spallation of oxidized fibers and clusters from the surface. This can be clearly observed in Movie S1 (Supporting Information) (tests 1 and 2). Although the reaction‐limited tests show minimal fiber elevation change over time, significant flattening begins to occur at approximately 100 s between 500–1400 μm, indicating early signs of structural collapse as volumetric oxidation weakens the porous material.

### 2.5 Modeling of Microscale Oxidation

**Figure 5:**
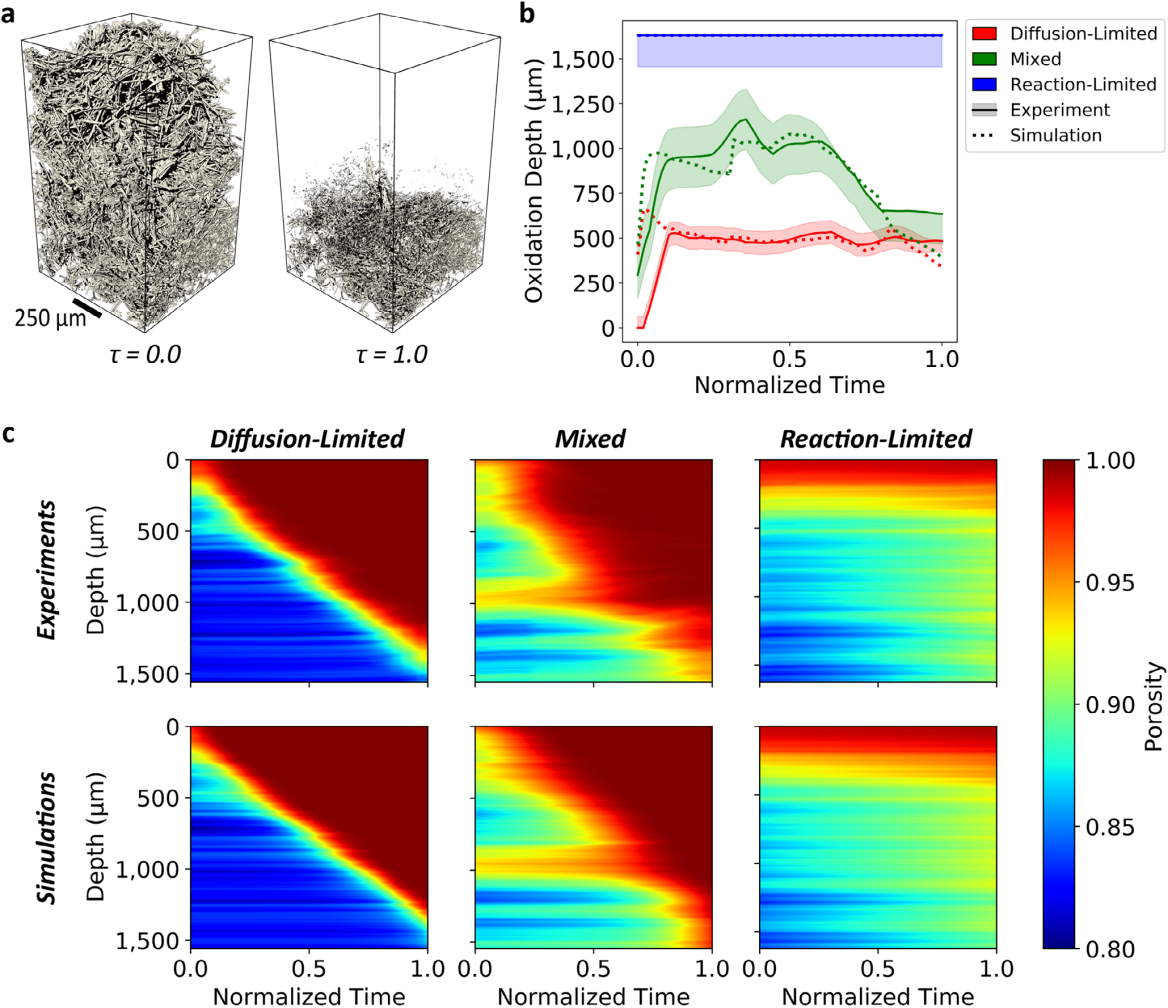
Microscale modeling of oxidation regimes. a) Renders of sample domain at the start (left) and end (right) of an oxidation simulation in PuMA, where τ is normalized time ranging from zero to one. b) Estimated oxidation depth of simulated data from PuMA and experimental data (*cf*. Section S1.7, Supporting Information for discussion of uncertainty). In both simulated and experimental cases, image data was cropped and scaled to identical dimensions (600 × 600 × 1008). c) Slice‐wise average porosity evolution over time for simulated and experimental data.

The oxidation regimes observed in this study were predicted in previous modeling work on carbon fiber materials.^[^ ^28^, ^29^, ^30^ ^]^ The model was based on earlier theoretical developments by Lachaud et al.,^[^ ^50^ ^]^ who demonstrated the ability to predict steady‐state roughness of carbon/carbon composites based on reactivity contrast between weakest and strongest reactive phases, and the nondimensional Sherwood number. In **Figure** **5** we show a comparison between the model, implemented in PuMA, and the measurements for test 1–3. The simulations use the first μ‐CT dataset collected in situ as the initial condition, and the Thiele numbers quantified in Figure 3e as input parameters. Oxidation depth and space‐time maps of porosity development are shown in Figure 5b,c, respectively. Note that, because the model only captures a steady state behavior, comparison is performed in normalized time. Excellent agreement between the simulations and the experimental measurements is demonstrated, with some discrepancies in transient regions: at high Thiele numbers, in the diffusion‐limited regime, the model predicts a narrow oxidation region close to the surface, while at low Thiele number, oxidation spans over the whole domain depth. The analysis confirms the effectiveness of Thiele number in capturing the microstructure evolution; however, time‐accurate predictions of material degradation would require higher‐fidelity finite‐rate models for the gas‐material interaction, such as those that have emerged in the recent ablation literature.^[^ ^2^, ^27^ ^]^

## 3 Conclusion

We have demonstrated the application of in situ μ‐CT to resolve the high‐temperature aerobic degradation of carbon fibers at the microscopic scale. The presented data provide key insights into the oxidation phenomenon, including direct quantification of porosity, microstructure evolution, and carbon mass loss rates. The experiments spanned regimes where material reactivity far exceeds diffusion or, conversely, where mass transport dominates over chemical reactions. Such information is invaluable for advancing modern ablation models, enhancing heat shield performance and tailoring materials to specific operational conditions.

The 4D description of the material microstructure enabled predictions of how effective properties evolve in space and time across different regimes. Currently, no other method provides access to this level of detail, which is critical for modeling the ablation response of composites under extreme aerothermal conditions. State‐of‐the‐art material response models rely on simplified interpolations between two experimental states – virgin and degraded – to track the changes in effective properties. These models fail to capture key effects such as the evolution of porosity imparted by in‐depth degradation. The effective property and oxidation profiles resolved with 4D imaging provide benchmarks for validating existing ablation solvers^[^ ^51^, ^52^ ^]^ and unified flow‐material models^[^ ^53^ ^]^ that capture in‐depth oxidation. We anticipate that these data will drive advancements in finite‐rate carbon oxidation models by incorporating surface area and porosity changes during oxidation, leading to improved bulk‐scale prediction capabilities across a wide range of flight conditions.

Our experiments highlight the importance of abating oxidation, even at relatively low temperatures with seemingly benign reaction rates, to preserve structural integrity and control reactant transport within the structure. Compositional strategies – such as aerogel resin impregnation, ceramic coatings, or nanostructural modifications – are adopted in fielded TPS materials to inhibit oxidation and enhance performance.^[^ ^54^ ^]^ With the ability to predict oxidation regimes and in‐depth degradation, new material designs could explore tailored densities, porosities, and optimized modifications for specific operational conditions or locations in a material stack. The design space is virtually unlimited. Controlled transport properties and anisotropy could be achieved by exploiting and scaling advances in additive manufacturing of carbon microstructures.^[^ ^55^, ^56^, ^57^ ^]^

While focused on TPS materials for hypersonics, the methods that we have developed in this work are broadly applicable to porous media with evolving porosities and phases, such as those found in chemical deposition, carbon capture and storage, and sedimentation processes, to name a few. Combining time‐resolved tomography with image‐based simulations and spatial filtering will lead to closure models for volume‐average simulations that accurately account for local variations in porosity.

Investigating composite materials that incorporate pyrolyzing resins is a natural extension of this work. The ability to capture rapid degradation processes – demonstrated here with time resolutions of approximately one event per second – paves the way for exciting advancements in the study of materials under extreme aerothermal environments. Continued improvements in μ‐CT acquisition and reconstruction techniques, combined with learning‐based data augmentation, will enable the exploration of more aggressive and faster degradation mechanisms, such as material interactions with atomic oxygen, carbon sublimation, and even coupled thermo‐chemo‐mechanical degradation phenomena.

## 4 Experimental Section

### In situ μ‐CT

Experiments were conducted using a custom‐built high‐temperature oxidation apparatus developed at the Advanced Light Source (ALS), consisting of an array of six confocal 150 W halogen infrared lamps with ellipsoidal reflectors and a controlled‐atmosphere reactor capable of 360° rotation (shown in Figure 1a,c). The oxidation reactor, shown in Figure 1b, comprises a 3 mm internal diameter (ID) fused quartz capillary, holding a 10 mm‐long sample by interference fit at one extremity. The lower end of the capillary tube was inserted into an aluminum ball/socket support for repeatable mounting in the chamber. An inlet gas supply line connected to the ball/socket enabled controlled mass flow through the sample chamber. The capillary tube was positioned such that the micro‐plug sample was centered on the lamp array focal point, providing radiative heating to the sample with a uniform hot‐region diameter of approximately 10 mm. High purity air was supplied from gas cylinders, providing O_2_ reactants to the sample, while helium was used as an inert atmosphere to flush oxidants from the reactor and impede oxidation before each test as the sample was brought to its steady‐state temperature.

All μ‐CT data was collected in the TOMCAT beamline X02DA at the Swiss Light Source (SLS) in Villigen, Switzerland. The high‐temperature reactor was placed in the path of the synchrotron X‐ray beam with a sample‐to‐scintillator distance of approximately 27.5 cm. A broad‐spectrum beam filtered using a 5 mm glassy carbon filter with a peak energy around 24 keV was utilized, and a high numerical aperture (NA) optical array and the GigaFRoST detection system were employed for all scans.^[^ ^16^ ^]^ Each radiograph was taken using an exposure time of 1 ms, a 100 μm thick cerium doped lutetium aluminum garnet (LuAG:Ce) scintillator, and a 10× lens from Optique Peter, resulting in an effective pixel size of 0.81 μm. For each tomography, 1000 projections were collected over a 180° rotation, with approximately 1 s between scans. A total of nine experiments were conducted at target lamp currents of 18, 15, and 13 A, and target pressures of 101.3, 26.7 , and 2.7 kPa (Table S1, Supporting Information).

### Sample Material

Samples were extracted from billets of a commercial material, trade named FiberForm (Fiber Materials, Inc., Biddeford, ME, USA). FiberForm is a carbon‐bonded carbon fiber insulator used in TPS material applications, most notably as substrate of NASA's Phenolic Impregnated Carbon Ablator (PICA).^[^ ^29^ ^]^ The material is made from a slurry of rayon‐derived carbon fibers, phenolic resin, and water, pressed along a specified direction *z*, and subsequently carbonized to high temperatures. The process leads to a low‐density (≈180 kg m^−3^), rigid, transverse isotropic structure, with preferential fiber alignment with the *xy*‐plane, and low thermal conductivity in the through‐thickness (TT) direction *z*. Previous characterizations have found a ±20° average fiber elevation with respect to the *xy* in‐plane (IP) direction.^[^ ^8^, ^9^ ^]^

Each sample was extracted by driving the thin‐walled 3 mm ID capillary tube into a FiberForm tile, in the TT direction. The quartz tube containing the sample was then placed in the vacuum chamber of the oxidation reactor, allowing the sample to be suspended and centered in the focal point of the radiative lamps. Assuming an ideal extraction, this process aligned the TT direction of the material with the rotation axis of the tomographic stage. Notably, this method of sample extraction could lead to increased fiber packing, resulting in reduced material porosity and elevated tortuosity (Table S3, Supporting Information). To match initial porosity across samples, pre‐test X‐ray screening may be utilized to ensure consistency in virgin material properties.

### Image Processing

The acquired radiography data was reconstructed using the Cori supercomputer at the National Energy Research Scientific Computing Center (NERSC). A custom Python script utilizing the gridrec algorithm from the TomoPy package^[^ ^58^ ^]^ was used for rapid and parallelized reconstruction of each dataset. Final 32‐bit reconstructions were converted to 8‐bit to simplify data handling. Due to the large overlap in void and fiber voxel intensity, reconstructed data was segmented using deep learning CNNs in ORS Dragonfly.^[^ ^35^ ^]^ Two‐class U‐Net deep learning models^[^ ^59^ ^]^ were trained for each dataset using ORS Dragonfly's Deep Learning Tool. For more details, see the Supporting Information.

## Conflict of Interest

The authors declare no competing interests.

## Supporting information

Supporting Information

Supplemental Video 1

Supplemental Video 2

Supplemental Video 3

## Data Availability

The reconstructed X‐ray tomography datasets, trained models, and corresponding segmentations discussed in this study are publicly available at https://doi.org/10.18126/nfkd‐mc22.
