# Supplementary material for: Carbon Fiber Oxidation in 4D
Source: Adv Mater. 2025 Jul 14;37(42):2502007. doi: 10.1002/adma.202502007 (PMC12548517; doi:10.1002/adma.202502007)
Supplement: Supplementary file 1 — Supporting Information [file ADMA-37-2502007-s002.pdf]

ADVANCED MATERIALS

Supporting Information

for *Adv. Mater.*, DOI 10.1002/adma.202502007

Carbon Fiber Oxidation in 4D

Benjamin M. Ringel, Federico Semeraro, Joseph C. Ferguson, Harold S. Barnard, Bruno Dias, Christian M. Schlepütz, Edward S. Barnard, Sam Schickler, Kara Levy, Shawn Shacterman, Talia Benioff-White, Julian Davis, Alastair A. MacDowell, Dilworth Y. Parkinson and Francesco Panerai**

Supporting Information for Carbon Fiber Oxidation in 4D

Benjamin M. Ringel*, Federico Semeraro, Joseph C. Ferguson, Harold S. Barnard, Bruno Dias, Christian M. Schlepütz, Edward S. Barnard, Sam Schickler, Kara Levy, Shawn Shacterman, Talia Benioff-White, Julian Davis, Alastair A. MacDowell, Dilworth Y. Parkinson, and Francesco Panerai*

*Corresponding authors. Emails: bringel2@illinois.edu, fpanerai@illinois.edu.

Contents

S1 Supporting methods **2**
S1.1 Selection of experimental conditions 2
S1.2 Image segmentation 3
S1.3 Mass loss rate and oxidation depth 4
S1.4 Reaction probability and Thiele number 5
S1.5 Effective material properties 7
S1.6 Visualization of material evolution 9
S1.7 Uncertainty 10

S2 Quantification of limiting oxidation regimes **10**
S2.1 Test conditions 10
S2.2 Porosity, mass loss, and oxidation depth 11
S2.3 Specific surface area 15
S2.4 Measured and computed quantities 17

S3 Anisotropy of effective properties **18**

Movies **21**

References **21**

S1 Supporting methods

S1.1 Selection of experimental conditions

Pressure and temperature conditions were selected based on calculated Thiele regimes for atmospheric entry trajectories and constraints imposed by the optimal spatiotemporal tomography resolution under achievable conditions.

To assess the flight relevance of potential test conditions, the post-shock temperature and pressure were computed along a low Earth orbit (LEO) lifting trajectory [1] and superorbital sample return trajectory [2, 3] using a 1D nonequilibrium stagnation line approximation [4], assuming a vehicle nose radius of 0.3 m and radiative equilibrium at the wall. Thiele numbers (Φ), shown in Figure S1, were then calculated for the reactive post-shock flow within a porous ablator throughout each trajectory using a material porosity of $\phi = 0.9$, a tortuosity of $\eta = 1.25$, a specific surface area of $s_f = 5 \times 10^4$ m^{-1}, and a characteristic length of $L = 1{,}632.2$ µm, matching L used in this study. Note that lower temperatures and pressures – and thus lower Thiele numbers – will occur along aft portions of the heat shield (*e.g.*, flanks and shoulders). Experimental conditions were chosen to target Thiele numbers along these trajectories at or near regions of extreme aerothermal loads ($\Phi = \mathcal{O}(10^{-1} - 10^{1})$)

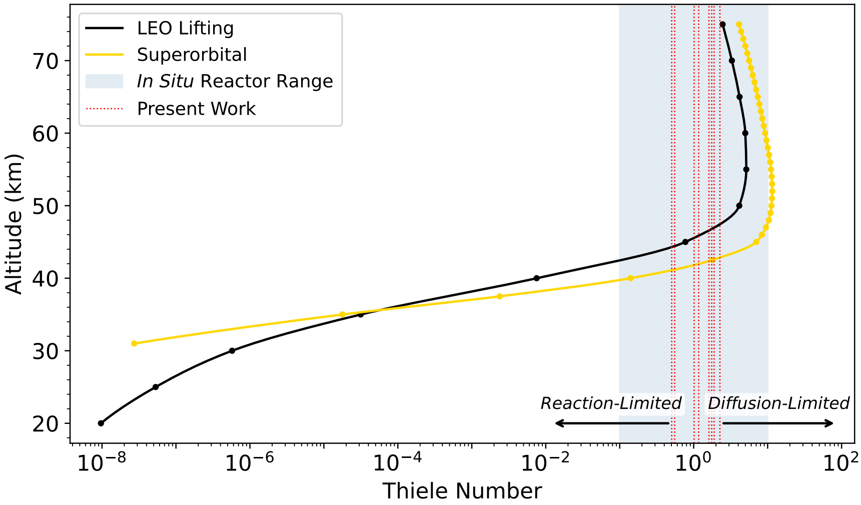

Figure S1: Estimated Thiele number profiles for a LEO lifting entry and superorbital entry compared against the expected range attainable in the *in situ* reactor (shaded region) and values calculated in the present work. Points denote locations along each trajectory where stagnation line computations were conducted.

and spanning the three regimes of interest: reaction-limited ($\Phi \ll 1$), diffusion-limited ($\Phi \gg 1$), and mixed ($\Phi \approx 1$). We refer the reader to Section S1.4 for details on Thiele number calculations.

A trade-off between estimated sample degradation rate and spatial resolution constrained accessible Thiele regimes within this flight-relevant envelope. To resolve salient features of the material, such as carbon fiber cross sections, a pixel size of less than 1 μm was required. At this spatial resolution, the maximum achievable acquisition rate of 1 Hz was chosen to minimize undersampling and spatially distorted data. Informed by prior degradation rates measured in oxygen [5], an upper temperature bound of 1,500 K was selected, a value sufficient to achieve diffusion-limited oxidation without exceeding the temporal resolution of the system. In the same manner, a lower temperature bound of ~900 K was established, representing the minimum temperature at which the facility could induce measurable mass loss over practical timescales under reaction-limited conditions. These upper and lower temperature bounds corresponded to lamp currents of 18 A and 13 A, respectively. The final range of attainable Thiele numbers was expanded by applying the selected temperatures over three pressures spanning the full range of experimentally accessible values. The specific temperatures and pressures measured in each test are provided in Section S2.1.

S1.2 Image segmentation

Due to significant overlap in void and fiber voxel intensity, two-class convolutional neural networks (CNNs) were trained in ORS Dragonfly's Deep Learning Tool [6] for enhanced image segmentation. Widely used in biomedical and scientific imaging and known for efficient training and application, a standard two-dimensional U-Net architecture was selected [7]. The network consists of four encoder stages and four decoder stages, with skip connections linking each encoder stage to its corresponding decoder stage. Each encoder stage includes two 3×3 convolutional layers with rectified linear unit (ReLU) activations, followed by a 2×2 max pooling operation for downsampling. Each decoder stage consists of a 2×2 upsampling operation, followed by concatenation with the corresponding encoder feature map and two subsequent 3×3 convolutions with ReLU activations. A final 1×1 convolutional layer produces the output image, assigning each pixel to a fiber or void class.

Training data were selected from a subset of 45 slices, drawn from three timepoints at the beginning, middle, and end of each experiment. At each of these timepoints, 15 slices were extracted to represent regions from the top, middle, and bottom of the sample, ensuring spatial and temporal diversity. Of these 45 slices, between 5 and 20 full slices were manually annotated depending on data contrast and noise. Additional partial-slice annotations were performed across the subset of each test, targeting regions with low contrast, high noise, or image artifacts. To expand the effective training dataset, augmentation was performed on all annotated images; data augmentation

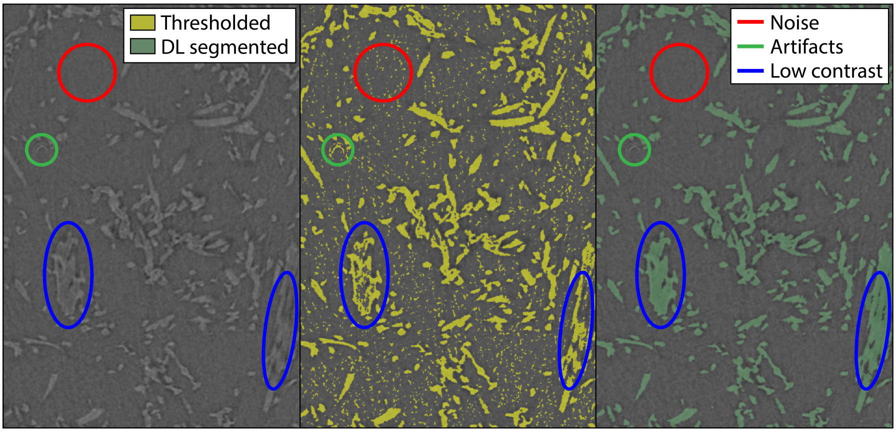

Figure S2: Demonstration of deep learning (DL) segmentation versus classic thresholding. Acquired CT data exhibits a strong overlap in voxel intensity in the void and fiber phases, making segmentation with thresholding challenging (center). Training a deep neural network on manually segmented data allows for advanced feature recognition and significantly more accurate semantic segmentation (right).

included flipping (vertical and horizontal), 180° rotation, 2° shearing, and random scaling between 90-110% of full-scale. Of this training dataset, 20% of images were reserved for validation. Model performance was evaluated using Dice coefficient and validation loss, yielding average final values of 0.978 and 0.022, respectively, across nine independently trained models, demonstrating high segmentation accuracy on the validation set.

A custom Python script was developed to apply these models to each timepoint using Dragonfly artificial intelligence segmentation libraries, with resulting segmentations output as binary images. The resulting 4D binary datasets can then be used to measure in-depth time-resolved properties, such as sample porosity, and allows for visualization of each test (see Supporting Movies).

As shown in Figure S2, a simple binary threshold yields a segmentation replete with noise in the void space, with image artifacts being segmented into the fiber class and regions of low contrast fiber being segmented into the void class. The trained CNN is able to better distinguish between void and fiber while ignoring image artifacts and void noise, leading to a significantly improved segmentation.

S1.3 Mass loss rate and oxidation depth

Following segmentation, a single experiment consists of a time series of three-dimensional (3D) binary arrays, with fiber voxels assigned the value one and void voxels assigned zero. Porosity ϕ of a the sample is defined as the ratio of void voxels to total voxels in the sample region of the image (*i.e.*, masked voxels

at the edges of the image are excluded). This can be expressed mathematically as

$$\phi = 1 - \frac{N_f}{N_s} \tag{1}$$

where N_f is the number of fiber voxels and N_s is the total number of voxels in the sample region. *I.e.*, porosity expresses the volume fraction of the sample occupied by void space.

Sample oxidation depth is defined as the in-depth distance between the sample surface and the deepest position where oxidation is measurably occurring, quantifying how far oxidation is penetrating the porous material at a given time. This quantity was measured using time-resolved change in depth-wise average porosity to find the surface of the sample and the absolute depth of oxidation. Absolute oxidation depth, defined as the distance from the top of the sample domain to the deepest point of measurable oxidation, was determined by computing change in porosity over time using a forward finite difference; for a given timepoint, the position in-depth where porosity is changing above a measured noise threshold is flagged as the absolute oxidation depth. The distance between the surface and the absolute oxidation depth yields oxidation depth at that point in time. The aforementioned noise threshold was measured at the beginning of each experiment in a virgin region of the sample domain, capturing temporal variation in porosity change due to segmentation error and fiber movement. By setting the oxidation threshold above this measured value, changes in porosity affecting the measurement of oxidation depth are isolated to mass loss from oxidation.

Mass flux $\dot{m}$ (where positive values indicate a decrease in mass) for a given timepoint was calculated as

$$\dot{m} = \rho s^3 N_s \frac{\Delta\phi}{\Delta t} \tag{2}$$

where ρ is the density of carbon fiber, s is voxel length, $\Delta\phi$ is the change in porosity from one timepoint to the next, and Δt is the time between tomographies. A density of ρ = 1,800 kg/m^3 was used with an uncertainty of ±200 kg/m^3 [8]. Performing this computation for each slice of data along the z axis at each timepoint yields depth-wise mass loss rate evolution (Figure S4).

S1.4 Reaction probability and Thiele number

Reaction probability of carbon and molecular oxygen is given by

$$\epsilon_{\mathrm{O}_2} = \frac{J_{\mathrm{C}}}{J_{\mathrm{O}_2}} \tag{3}$$

where J_{C} is the flux of carbon atoms out of the sample domain and J_{O_2} is the incoming flux of molecular oxygen. Carbon element flux can be calculated as

$$J_{\mathrm{C}} = \frac{\dot{m}}{m_{\mathrm{C}} A_{\mathrm{ox}}} \tag{4}$$

where $\dot{m}$ is the measured steady-state mass loss rate, m_{C} is the atomic mass of carbon, and A_{ox} is the measured steady-state oxidized surface area. Oxygen molecular flux is described as

$$J_{\mathrm{O}_2} = \frac{1}{4} \frac{p_{\mathrm{O}_2}}{kT} \bar{\nu} \tag{5}$$

where p_{O_2} is the partial pressure of oxygen for each experiment, k is the Boltzmann constant, T is the measured temperature, and $\bar{\nu}$ is the mean molecular velocity, which is given by

$$\bar{\nu} = \left(\frac{8RT}{\pi M_{\mathrm{O}_2}} \right)^{1/2} \tag{6}$$

where R is the universal gas constant, T is the measured temperature, and M_{O_2} is the molar mass of molecular oxygen. Reaction probability was computed for experiments in sample sets A, B, and C, and results can be seen in Figure 3d and Table S3.

Competition between species diffusion and reaction yield the varying oxidation regimes observed in this work. A way of quantifying this diffusion-reaction competition in porous media is using the dimensionless quantity Thiele number, which can be described as

$$\Phi = \frac{L}{[D_{\mathrm{eff}}/(s_f k_f)]^{1/2}} \tag{7}$$

where L is the length of the sample domain, D_{eff} is the effective diffusivity, s_f is the specific surface area of the fibers, and k_f is the fiber reactivity. When reactivity is high relative to species diffusive flux, Thiele number will be high, and a diffusion-limited regime will be observed. Alternatively, when reactivity is low relative to diffusivity, Thiele number will be low, and a reaction-limited regime will be observed. For an isotropic porous material, effective diffusion coefficient is described as

$$D_{\mathrm{eff}} = \frac{\phi D_{\mathrm{ref}}}{\eta} \tag{8}$$

where ϕ is the bulk porosity of the sample, D_{ref} is the reference diffusivity [9], and η is the measured bulk tortuosity of the material. Bulk porosity ϕ was measured directly using the first segmented image of each experiment and reference diffusivity D_{ref} was taken as the mixture averaged diffusion coefficient calculated in Mutation++ [10] at the measured temperature and pressure of

each experiment. Bulk tortuosity η was computed in PuMA for the first image domain in each experiment. Specific surface area is defined as

$$s_f = \frac{A_f}{V} \tag{9}$$

where A_f is the surface area of the fibers and V is the volume of the sample domain (fiber and void). Finally, fiber reactivity can be defined as

$$k_f = \frac{\epsilon_{O_2}}{4}\,\bar{\nu} \tag{10}$$

Thiele number was computed for all experiments in sample sets A, B, and C. Results can be seen in Figure 3e and Table S3.

S1.5 Effective material properties

Determination of material properties in porous multi-phase materials, such as FiberForm, is a multiscale problem. To compute effective properties for a given volume of the material, one must solve the governing equations at the microscale and use up-scaling techniques to obtain meso- or macroscale values for effective properties [11]. The most common up-scaling technique is the volume-averaging method [12], which consists of averaging the pore-scale equations and physical quantities via spatial convolution with a filter function. With this approach, one must consider the filter's kernel and size of the representative elementary volume (REV) to obtain well-behaved average properties.

The effective properties can be derived from a unit cell for periodic and homogeneous porous medium. If the problem is linear, the closure properties can be derived from mapping variables that are solved as a boundary-value problem in a unit REV [13, 14]. Using this methodology, Whitaker [15, 16] derived the permeability and Forchheimer tensor based on Darcy's law, and Carbonell and Whitaker [17] derived effective thermal conductivity and tortuosity coefficient for a heat and mass transfer problem. Conversely, if the porous structure is highly complex and the problem is non-linear, these properties can only be derived from porous scale simulations.

In the present work, the approach of Breugem and Dias *et al.* [18, 19] is utilized to derive effective properties such as thermal conductivity, permeability, and tortuosity. The approach involves solving the heat conduction, Stokes, and diffusion equation at the pore-scale and volume averaging the physical quantities using a spatial convolution. Afterward, the macroscopic equations for effective properties are evaluated using the averaged physical quantities at each point in space.

Let ψ^v be a generic quantity at the pore-scale such as temperature, velocity, and pressure. In the void-phase these quantities are defined as [20]

$$\psi_v(\mathbf{r}, t) = \psi^v(\mathbf{r}, t)\gamma_v(\mathbf{r}, t) \tag{11}$$

where the γ_v is the phase indicator [21, 22] , *i.e.*,

$$\gamma_v(\mathbf{r}, t) = \begin{cases} 1, \ \mathbf{r} \text{ in void-phase} \\ 0, \ \text{Otherwise} \end{cases} \tag{12}$$

where $\mathbf{r}$ is the position vector.

The phase average is the convolution product of the filter weighting function G and the quantity ψ in the void-phase yielding,

$$\langle\psi\rangle^{\mathbf{V}}\Big|_{\mathbf{x}} \equiv G * \psi_v\Big|_{\mathbf{x}} = \frac{1}{V}\int_{\mathbb{R}^3} G(\mathbf{x}-\mathbf{r})\,\psi^v(\mathbf{r},t)\,\gamma_v(\mathbf{r},t)\,\mathrm{d}^3\mathbf{r} \tag{13}$$

where $\mathbf{x}$ is the REV center's position vector and V is the volume. A top-hat filter weighting function [12, 22, 23] is defined as,

$$G(\mathbf{x}-\mathbf{r}) = \begin{cases} 1, \ |\mathbf{x}-\mathbf{r}| < r_0 \\ 0, \ |\mathbf{x}-\mathbf{r}| \geq r_0. \end{cases} \tag{14}$$

where r_0 is the minimum size that allows a well-behaved REV.

The REV porosity is defined as

$$\phi \equiv G * \gamma_v\Big|_{\mathbf{x}} = \frac{1}{V}\int_{\mathbb{R}^3} G(\mathbf{x}-\mathbf{r})\,\gamma_v(\mathbf{r},t)\,\mathrm{d}^3\mathbf{r} = \frac{V_v}{V} \tag{15}$$

where V_v is the volume of the void space. The average of properties in the void space of the material, known as the intrinsic average, can be written as

$$\langle\psi\rangle\Big|_{\mathbf{x}} \equiv \frac{G * \psi_v|_{\mathbf{x}}}{G * \gamma_v|_{\mathbf{x}}} = \frac{1}{V_v}\int_{\mathbb{R}^3} G(\mathbf{x}-\mathbf{r})\,\psi^v(\mathbf{r},t)\,\gamma_v(\mathbf{r},t)\,\mathrm{d}^3\mathbf{r}, \tag{16}$$

leading to the following relation between the phase and intrinsic average,

$$\langle\psi\rangle^{\mathbf{V}} = \phi\,\langle\psi\rangle\,. \tag{17}$$

PuMA simulations were conducted on downsampled regions of tests 1, 2, and 3. All timepoints of each test were cropped to a shape of 1,200×1,200×2,016, then resampled by a factor of 0.5 to decrease simulation time, yielding a final domain shape of 600×600×1,008. From converged simulations at each point in time, 3D property fields with the same shape were convolved, resulting in intrinsic and phase average property arrays, which were used to compute final effective property fields. Valid convolutions were performed with a kernel of size 71×71×71 using a top-hat filter.

Effective thermal conductivity was calculated via the macroscopic heat-conduction equation, defined as

$$\langle q_i\rangle^{\mathbf{V}} = k_{\mathrm{eff},ii}\frac{\partial\,\langle T\rangle^{\mathbf{V}}}{\partial x_i} \tag{18}$$

where $k_{\text{eff},ii}$ is the effective thermal conductivity at position ii in the effective thermal conductivity tensor $\bar{\boldsymbol{k}}_{\text{eff}}$, q_i is the ith component of the heat-flux vector field, and T is the scalar temperature field. This work focuses only on the diagonal terms of effective property tensors (*i.e.*, the TT and IP quantities). In order to obtain the three components of the diagonal tensor, the heat-conduction equation is solved separately for each direction $i = 1, 2, 3$ at the pore-scale as shown in refs. [24–26]. Effective permeability was computed from the average Stokes equation [15, 18, 19] which can be written as

$$\mu \frac{\langle u_i \rangle^{\mathbf{V}}}{\mathrm{K}_{\text{eff},ii}} = \frac{\partial \langle p \rangle}{\partial x_i} \tag{19}$$

where $\mathrm{K}_{\text{eff},ii}$ is the effective permeability at position ii in the effective permeability tensor $\bar{\mathbf{K}}_{\text{eff}}$, u_i is the ith component of the velocity vector field, and p is the scalar pressure field. The macroscopic diffusion equation for species α is defined as

$$\langle J_i^\alpha \rangle^{\mathbf{V}} = D^\alpha_{\text{eff},ii} \frac{\partial \langle C^\alpha \rangle}{\partial x_i} \tag{20}$$

where D^α_{eff} is the effective diffusion coefficient at position ii in the effective diffusivity tensor $\bar{\boldsymbol{D}}^\alpha_{\text{eff}}$, J_i^α is the ith component of the diffusive flux vector field, and C^α is the scalar concentration field. Finally, the tortuosity factor is defined as

$$\eta_{\text{eff},ii} = \phi \frac{D_{\text{ref}}}{D^\alpha_{\text{eff},ii}} \tag{21}$$

where $\eta_{\text{eff},ii}$ is the effective tortuosity factor at position ii in the effective tortuosity tensor $\bar{\boldsymbol{\eta}}_{\text{eff}}$.

Following computation of effective thermal conductivity, permeability, and tortuosity (each direction being represented with a 3D scalar field), the slice-wise average properties were computed for each timepoint to get in-depth effective properties over time in a diffusion-limited, mixed, and reaction-limited regime. Results are shown in Figure 4, and observations of anisotropic material behavior are discussed in Section S3.

S1.6 Visualization of material evolution

Contour plots shown throughout this work (*e.g.*, Figures 2, 4, and 5) visualize the evolution of 4D dataset properties across depth and time via slice-wise averaging. For each timepoint, the 3D material property domain is reduced to a 1D profile by computing the mean property value at each 2D slice along the in-depth (z) direction. Repeating this process across all timepoints yields a 2D property field, visualized as a contour map, where a vertical line represents a depth-resolved property distribution for a single timepoint, and a horizontal line represents the temporal evolution of properties at a fixed depth.

S1.7 Uncertainty

With error in segmented images often relegated to the interface between fiber and void space due to diffuse contrast and fringe artifacts at fiber edges, uncertainty in segmented data was determined by performing a 2D dilation and erosion along the xy-plane of each dataset with a 3×3 kernel. This process yielded two additional datasets for each experiment (dilated and eroded), allowing for the estimation of upper and lower uncertainty bounds for properties such as porosity (±3%), tortuosity (±5%), specific surface area (±6%), and mass loss rate (±16%), which also considered an uncertainty in fiber density of ±200 kg/m^3.

The noise threshold used to compute oxidation depth (see Section S1.3) carries uncertainty due to temporal variation in porosity measurements. To ensure conservative estimates, this uncertainty was applied to the maximum measured absolute oxidation depth for each test, resulting in bounds of up to ±15% (Figures 3b, 5b, and S5). These bounds were then used to determined the corresponding uncertainty in oxidation fraction (Figure 3c). Note that estimated uncertainty is constrained by the physical boundaries of the domain (0 to 1,632.2 μm), resulting in asymmetric uncertainty bounds when the measured oxidation reaches the bottom of the sample.

To propagate parameter uncertainties to reaction probability, we performed a parametric sweep of all combinations of oxidized surface areas and mass loss rates derived from perturbed segmentations and variations in fiber density. The resulting upper and lower uncertainty bounds (Figure 3d) reflect the combined effects of material property and surface area uncertainties. These bounds, along with porosity and tortuosity values from the perturbed datasets, were then used to compute corresponding uncertainty estimates for Thiele number (Figure 3e).

Sources of uncertainty for computed effective properties (Figure 4) include segmentation and modeling errors. In the case of effective thermal conductivity, uncertainty in constituent properties assigned to the fibers also contribute. Although these uncertainties were not explicitly quantified for the effective property computations, we expect that variations arising from material heterogeneity exceed the error introduced by these sources.

S2 Quantification of limiting oxidation regimes

S2.1 Test conditions

As shown in Table S1, tests were conducted at three lamp currents and three pressure conditions for a total of nine experiments. Samples were divided into three sets, with each set containing experiments at a constant target pressure. Tests were conducted at target pressures of 101.3, 26.7, and 2.7 kPa in sample sets A, B, and C, respectively.

It can be seen in Table S1 that as lamp current increases, chamber temperature increases, and in most cases there is also a slight increase in pressure.

In addition, between sample sets A, B, and C, it can be seen that as pressure decreases between tests at constant lamp current there is an increase in temperature. This observed increase in temperature is due to a lower capacity of the fluid to absorb and dissipate heat as pressure declines.

Table S1: Measured lamp current (I), chamber temperature (T), and chamber pressure (P). All tomographies were collected at an acquisition frequency of 1 Hz.

Sample Set	Test #	I (A)	T (K)	P (kPa)
	1	18	1,320	100.5
A	2	15	1,060	100.0
	3	13	945	100.7
	4	18	1,375	28.5
B	5	15	1,185	22.1
	6	13	1,030	20.4
	7	18	1,445	3.0
C	8	15	1,220	2.4
	9	13	1,055	2.4

S2.2 Porosity, mass loss, and oxidation depth

In-depth porosity and mass loss rate evolution of all 4D μ-CT experiments can be seen in Figure S3 and Figure S4, respectively, where effects due to pressure and temperature variation are shown. In addition, measured oxidation depth of each experiment is shown in Figure S5.

Evolution of porosity over time for all three high-temperature cases (tests 1, 4, and 7) show similar characteristics, with differences due to changes in pressure. In test 1 at 101.3 kPa (Figure S3a, left), change in porosity is relegated to a small depth near the sample surface as the high fiber reactivity and relatively low diffusivity leads to a depletion of oxygen before reaching far into the sample domain. This surface-limited oxidation is also shown in Figure S4a (left), where a majority of mass leaves the sample domain within a narrow band, moving in depth over time as the sample surface recesses, and is further evidenced by a small oxidation depth shown in Figure S5 (left). Test 4, at a chamber pressure of 26.7 kPa (Figure S3b, left), shows similar features, exhibiting a broader affected depth where porosity increases in time when compared with test 1 at 101.3 kPa. This trend is enforced in the left plot of Figure S4b, which displays a wider depth of mass removal compared to that of test 1, and Figure S5 (center), which shows a slightly deeper measured oxidation depth for test 4 compared to test 1. The final high-temperature test (test 7) at a pressure of 2.7 kPa further demonstrates this pattern, with porosity evolution over time (Figure S3c, left) exhibiting an even greater affected

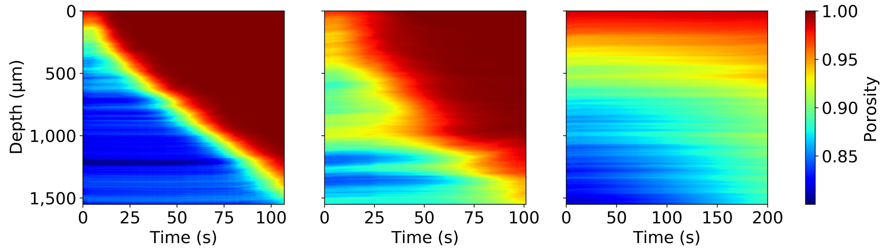

(a)

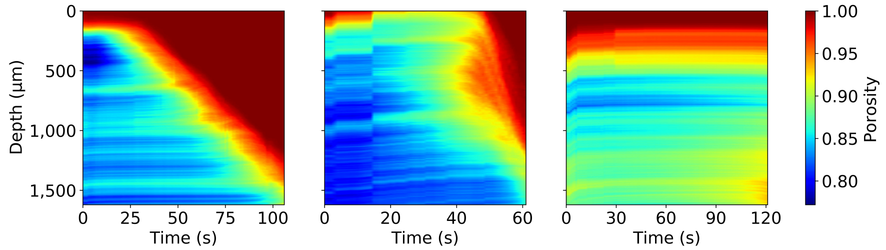

(b)

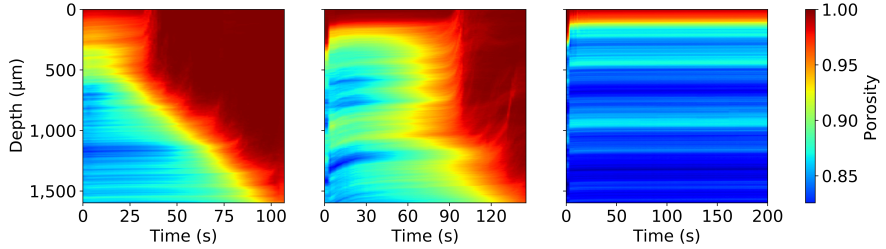

(c)

Figure S3: Measured porosity evolution of 4D μ-CT experiments at target pressures of (a) 101.3 kPa (tests 1, 2, and 3), (b) 26.7 kPa (tests 4, 5, and 6), and (c) 2.7 kPa (tests 7, 8, and 9) with diffusion-limited, mixed regime, and reaction-limited experiments shown from left to right, respectively.

depth, and mass loss rate over time once again showing a wider region of mass removal that remains near the surface as the sample recesses. It can also be seen in Figure S5 (right) that test 7 exhibits a significantly deeper oxidation depth compared to both tests 1 and 4. This increase in oxidation depth at lower pressures is due to an increase in diffusivity; even with a slight increase in measured temperature as pressure decreases (shown in Table S1), diffusion of reactants through the porous media increases significantly compared with the relatively constant reactivity of the carbon fibers, leading to a greater oxidation depth. While a decrease in chamber pressure does lead to an increase in oxidation depth, in all three diffusion-limited cases, sample oxidation resides

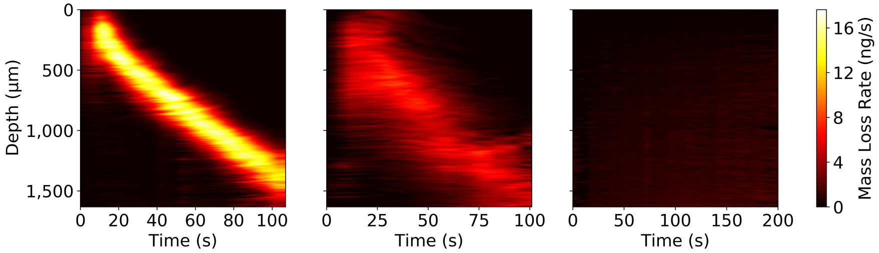

(a)

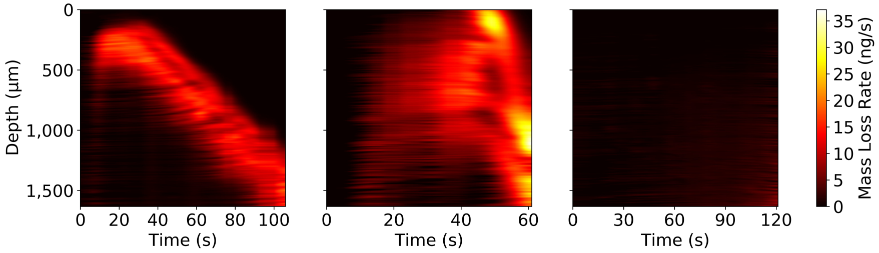

(b)

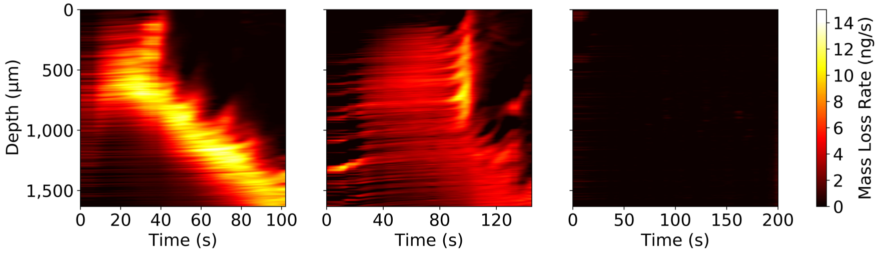

(c)

Figure S4: Measured mass loss rate of 4D µ-CT experiments at target pressures of (a) 101.3 kPa (tests 1, 2, and 3), (b) 26.7 kPa (tests 4, 5, and 6), and (c) 2.7 kPa (tests 7, 8, and 9) with diffusion-limited, mixed regime, and reaction-limited experiments shown from left to right, respectively.

near the surface, with sample mass removal moving in-depth as the sample recesses.

Medium-temperature tests (tests 2, 5, and 8) demonstrate a mixed oxidation regime in the center plots of Figure S3 and Figure S4, and measured oxidation depth can be seen in Figure S5. As shown if Figure S3a (center), for test 2 at 101.3 kPa, a significant depth of the sample is effected, showing broad porosity evolution in-depth over time. The center plot of Figure S4a shows, however, that while the depth of mass removal is wider than in the diffusion-limited case, mass is not being removed everywhere in the domain depth. In addition, it can be seen that compared to the diffusion-limited case in test 1

(Figure S3a, left), the magnitude of mass loss rate decreases while the depth of oxidation increases. This is because at a lower temperature and equivalent target pressure, carbon fiber reaction rate decreases while diffusivity remains approximately the same, allowing available oxygen to diffuse further into the sample before being fully depleted. Test 5 porosity evolution (Figure S3b, center) appears different compared to the higher pressure case in test 2, with a slight delay in noticeable porosity change, with the exception of sharp shifts in porosity due to the sample shifting in the apparatus. This upward sample shift can be seen in Movie S2 (center). In this video, it can be seen that once oxidation has begun, the top of the sample sees greater change in porosity than the bottom section of the sample. At around 50 s, the sample then structurally fails and slowly exits the image domain, yielding artificially high mass loss values shown in the center plot of Figure S3b. Note that the center plots of both Figure S3b and Figure S4b were cropped in time after the sample started to collapse, allowing for better dynamic range within the valid region of the test. The full test is available to view in Movie S2 (center). The final medium temperature test (test 8) at a chamber pressure of 2.7 kPa also shows complicated results when analyzing the center plots of Figure S3c and Figure S4c, where it is evident that the sample once again shifts in the apparatus at the beginning of the test, yielding sharp changes to porosity within the first 10 s. In addition, mass loss results (Figure S4c, center) and oxidation depth measurement (Figure S5, right) reveal that mass is being removed throughout the entire sample domain, and both porosity and mass loss demonstrates sample structure failure at the end of the test with large shifts in measured values after 90 s. From Movie S3 (center), it can be seen that porosity is indeed increasing everywhere in the image domain; however, it can also be observed that porosity increases at a greater rate at the top of the domain compared to the bottom. Movie S3 (center) also shows sample structural failure after 90 s. While depth of oxidation was shown to increase with decreasing pressure (as shown in Figure S5), in all medium temperature tests it was also shown that the rate of oxidation decreases as depth increases, with oxidation rate being greater at the sample surface compared to at the bottom of the oxidation depth.

For low-temperature experiments (tests 3, 6, and 9), oxidation was found to be limited by the reaction rate of the fibers across all pressures, as shown in the right-most plots on Figure S3 and Figure S4. For both test 3 (Figure S3a, right) and test 6 (Figure S3b, right), conducted at 101.3 kPa and 26.7 kPa, respectively, porosity can be seen to increase throughout the sample domain over the duration of the test. Due to low reaction rates, mass loss results shown in the right-most plots of Figure S4a (test 3) and Figure S4b (test 6) are low in magnitude at any given point in space compared to diffusion-limited and mixed regime tests. The effects of reaction-limited oxidation can be seen more clearly in Movie S1 (test 3) and Movie S2 (test 6). In these videos, it is shown that porosity increases slowly and evenly throughout the entire sample domain. Additionally, Movie S2 (right) demonstrates sample structural failure

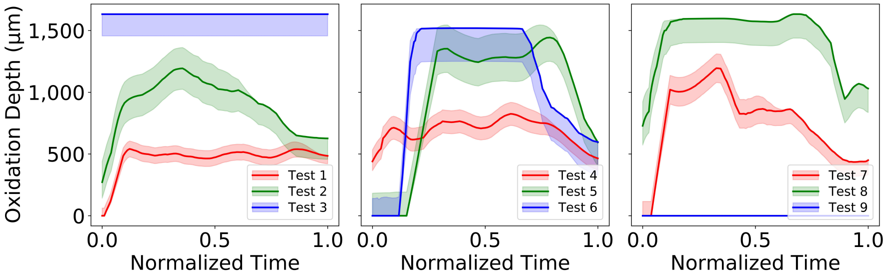

Figure S5: Measured oxidation depth over normalized time for sample set A (left), sample set B (center), and sample set C (right). Note that the full depth of the domain is 1,632.2 µm.

after 120 s. In both tests 3 and 6, measured oxidation depth exhibits full domain oxidation, as shown in Figure S5, with test 6 only yielding a measured depth slightly below the maximum value of 1,632.2 µm due to the sample surface not extending to the top of the image domain. Porosity evolution for Test 9 (Figure S3c, right), conducted at 2.7 kPa, shows a slight sample shift at the beginning of the test, followed by no change in porosity for the full 200 s experiment. This is further demonstrated in the right-most plot of Figure S4c, where no mass loss was measured throughout the domain, and can also be verified in Movie S3 (right), where porosity does not shift throughout the test. In each of these reaction-limited cases, temperature is low, and therefore fiber reactivity is low compared with mixed and diffusion-limited cases. With a low fiber reactivity and sufficiently high diffusivity, oxidation occurs evenly throughout the sample volume (if at all); there is never a scarcity of reactants, leading to fully volumetric oxidation.

S2.3 Specific surface area

Computed specific surface area (Eq. 9) can be seen be seen in Figure S6 for both the total sample domain and the estimated oxidation depth. For diffusion-limited tests (left), sample surface area begins to decline quickly, reaching a roughly linear steady-state rate of decrease. At the same time, oxidized surface area reaches a roughly constant steady-state as diffusion-reaction competition equilibrates. For test 1 at a target pressure of 101.3 kPa (Figure S6a, left), the oxidized area is less than that of the corresponding diffusion-limited tests (tests 4 and 7); with a lower diffusivity at higher pressure, oxidation depth is less (Figure S5), leading to a smaller oxidized area. Conversely, in the lower pressure case of test 7 (Figure S6c, left), the measured depth of oxidation is the greatest of all three diffusion-limited cases, leading to an increase in the fraction of available surface area oxidized, and in this case, leading to a full saturation of the sample domain approximately 65 s into the experiment, as evidenced by the equality of total and oxidized surface area. Note that in some

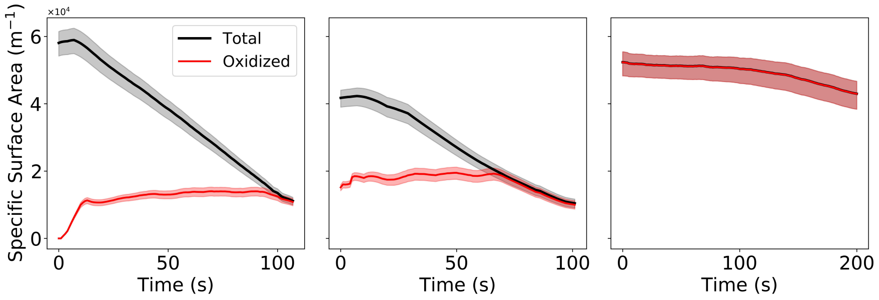

(a)

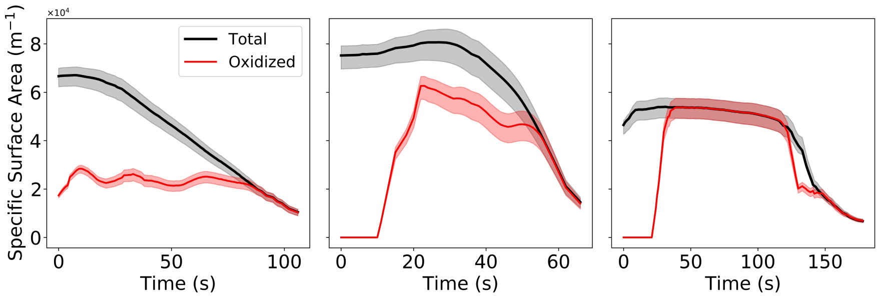

(b)

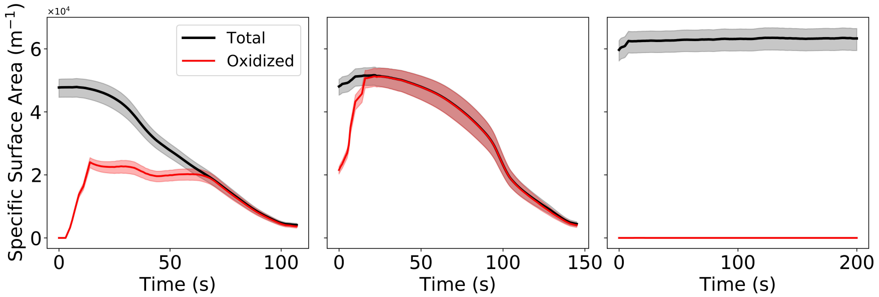

(c)

Figure S6: Measured specific surface area, defined as fiber surface area normalized by the sample domain volume, of the total sample domain and oxidized region of the sample for tests at target pressures of (a) 101.3 kPa (test 1, 2, and 3 from left to right, respectively), (b) 26.7 kPa (tests 4, 5, and 6 from left to right, respectively), and 2.7 kPa (tests 7, 8, and 9 from left to right, respectively). Uncertainty bands surrounding measurements were determined from perturbation of edge pixels in segmented data.

cases, measured total area increases due to slight upward shifts of the sample in the z-direction, causing more fiber area to enter the sample domain.

In mixed regime cases, the transition from surface-dominated oxidation to volumetric oxidation emerges. In both test 2 (Figure S6a, center) and test 5 (Figure S6b, center), oxidized surface area approaches a steady-state value that is greater than its respective diffusion-limited case before the domain is fully oxidized and total surface area matches oxidized surface area. In contrast, for test 8 (Figure S6c, center) at a target pressure of 2.7 kPa, a high diffusivity leads to oxidation throughout the sample domain over the majority of the experiment. Note the decrease in oxidized surface area in test 5 (Figure S6b, center) and the sudden decrease in total surface area near the end of tests 5 and 8 (Figure S6c, center), a result of sample structural failure, which can be seen in Movies S2 (center) and S3 (center).

Reaction-limited test 3 (Figure S6a, right) and test 6 (Figure S6b, right), conducted at target pressures of 101.3 and 26.7 kPa, respectively, show that oxidation occurred throughout the entire volume of the sample for the majority of the test, slowly decreasing sample surface area, and in the case of test 6, leading to sample collapse (shown in Movie S2, right). In test 9 (Figure S6c, right), due to low temperature and oxygen partial pressure, no significant change in surface area was observed.

S2.4 Measured and computed quantities

A summary of measured steady-state oxidation parameters and observed Thiele oxidation regimes can be seen in Table S2. Steady-state oxidation depth estimates show the same trend as shown in Figure 3c, exhibiting increasing oxidation depth with decreasing temperature (*i.e.*, decreasing reactivity) and decreasing pressure (*i.e.*, decreasing diffusivity). Note that in all reaction-limited cases, oxidation reaches the bottom of the domain, but that values for oxidation depth are not always at the maximum value of 1,632.2 μm due to the surface of the sample sitting below the top of the image domain. An increase in steady-state oxidized surface area with increasing temperature can also be seen; however, the measured oxidized surface area varies significantly

Table S2: Average steady-state oxidation depth (z_{ox}), oxidized surface area (A_{ox}), and mass loss rate ($\dot{m}$), as well as the observed oxidation regime of each test.

Sample Set	Test #	z_{ox} (μm)	A_{ox} (mm^2)	$\dot{m}$ (μg/s)	Regime
	1	485.5	35.14	6.176	Diffusion-limited
A	2	1,127.8	59.39	6.087	Mixed
	3	1,632.2	171.2	1.291	Reaction-limited
	4	757.9	79.56	9.416	Diffusion-limited
B	5	1,297.9	222.6	9.253	Mixed
	6	1,518.2	177.2	1.770	Reaction-limited
	7	843.4	68.60	7.314	Diffusion-limited
C	8	1,590.9	159.1	5.752	Mixed
	9	0.0	0.0	0.0	No oxidation

Table S3: Calculated material and fluid properties, including initial bulk material porosity (ϕ_{bulk}), initial bulk tortuosity (η_{zz}) in the z-direction, reference diffusivity (D_{ref}), effective diffusivity (D_{eff}), reaction probability of C and O_2 (ϵ_{O_2}), fiber reactivity (k_f), and Thiele number (Φ).

Sample Set	Test #	ϕ_{bulk}	η_{zz}	D_{ref} (m^2/s)	D_{eff} (m^2/s)	ϵ_{O_2}	k_f (m/s)	Φ
	1	0.858	1.31	3.27E-04	2.14E-04	3.25E-05	7.60E-03	2.26
A	2	0.898	1.22	2.30E-04	1.68E-04	1.71E-05	3.58E-03	1.75
	3	0.883	1.23	1.84E-04	1.32E-04	1.18E-06	2.33E-04	0.50
	4	0.858	1.40	1.24E-03	7.64E-04	7.90E-05	1.88E-02	1.89
B	5	0.854	1.37	1.25E-03	7.75E-04	3.31E-05	7.34E-03	1.17
	6	0.906	1.24	1.07E-03	7.83E-04	8.06E-06	1.66E-03	0.55
	7	0.901	1.22	1.31E-02	9.63E-03	7.05E-04	1.72E-01	1.61
C	8	0.897	1.27	1.20E-02	8.48E-03	2.68E-04	6.03E-02	1.01
	9	0.877	1.32	9.67E-03	6.44E-03	0.0	0.0	0.0

between pressure conditions at similar temperatures due to variation in sample porosity (shown in Table S3). For diffusion-limited and mixed regime cases in sample sets A and B, measured values of steady-state mass loss rate show strong agreement at their respective pressure condition. In each of these cases, reactants cannot reach the bottom of the domain before being fully consumed due to high fiber reactivity, meaning that for tests at the same pressure condition, approximately equivalent carbon mass is removed over time, even with varying depth of oxidation.

Computed bulk material and fluid properties for each experiment are shown in Table S3. It can be seen that initial bulk porosity varies between 0.854 and 0.906, which, as previously discussed, leads to variation in material properties, such as tortuosity. Trends exhibited in Figure 3d can also be seen, with an increase in reaction probability with increasing temperature, and pressure effects can be observed, with reaction probability increasing as pressure decreases. Values displayed in Figure 3e are also shown, quantifying the observed diffusion-reaction competition with high Thiele numbers in diffusion-limited cases and low Thiele numbers in reaction-limited cases. Note that for all Thiele number calculations, a sample length of L = 1,632.2 µm and an average initial specific surface area of s_f = 54,223 m^{-1} was used.

S3 Anisotropy of effective properties

PuMA conductivity computations were conducted in the x, y, and z-directions for all time points of test 1 (diffusion-limited), test 2 (mixed), and test 3 (reaction-limited). As described in the Methods section, during sample extraction, the z axis of the sample was aligned perpendicular to the FiberForm billet IP direction, meaning that the xy-plane of the sample ideally represents

the IP direction, while the z axis of the sample represents the TT direction. This allows for analysis of time-resolved effective conductivity calculations of both the IP and TT directions separately. Because FiberForm is transverse isotropic, conductivity results in the x and y directions were averaged to obtain the IP effective conductivity k_{IP}. TT effective conductivity k_{TT} was taken as the conductivity results in the z-direction.

Depth-wise evolution of TT effective thermal conductivity is compared to IP effective thermal conductivity in Figure S7. In the diffusion-limited case (test 1), there is a large difference in TT and IP effective thermal conductivity, with TT values in the virgin region of the material around 0.45-0.50 W/(m·K), while IP values are approximately 0.60-0.70 W/(m·K) at the same location. This trend can also be seen in the mixed regime case, with k_{TT} around 0.25 W/(m·K) and k_{IP} near 0.50 W/(m·K) at the bottom of the sample for the first 25 s of the experiment. The increase in effective thermal conductivity magnitude between the mixed case and diffusion-limited case is due to an increase in sample density, shown by lower initial porosity in diffusion-limited test (Figure S3a).

The trend of greater computed effective thermal conductivity in the IP direction compared to the TT direction is not observed in the reaction-limited case (test 3). While there are sections of the domain where effective thermal conductivity is slightly greater in the IP direction compared to the TT direction, for the majority of the test, effective thermal conductivity

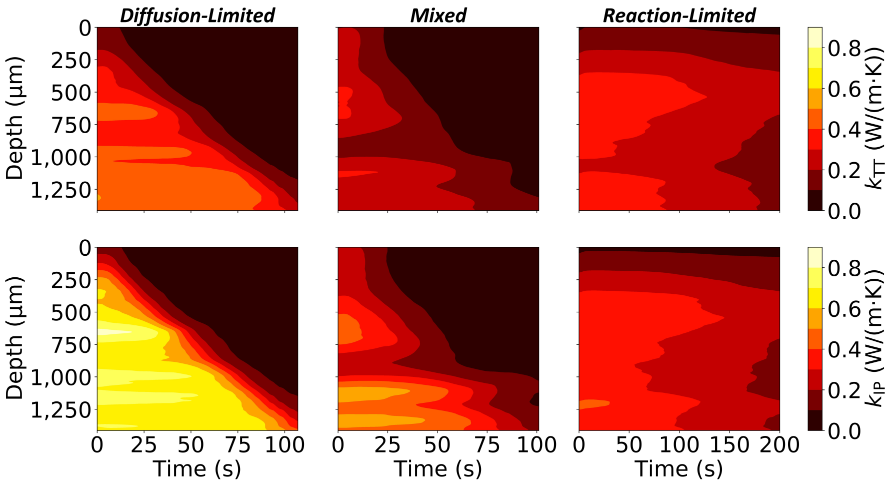

Figure S7: Comparison between TT and IP effective thermal conductivity over time for diffusion-limited (test 1), mixed (test 2), and reaction-limited (test 3) cases. Similar values in the TT and IP directions for the reaction-limited case suggest the IP direction of the material was not aligned with the xy-plane of the image domain for test 3.

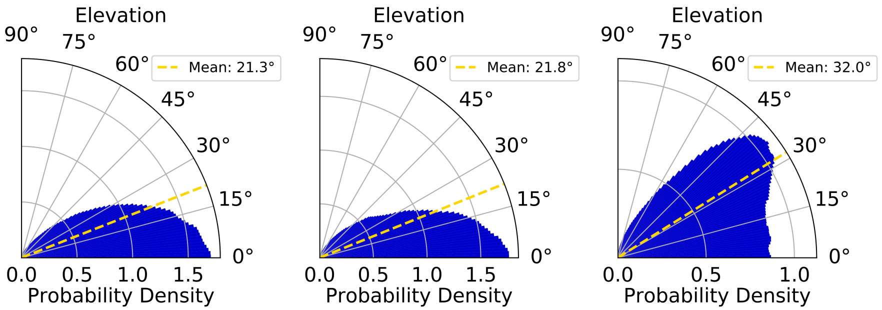

Figure S8: Probability density distributions for fiber elevation for the first time point in each experiment in sample set A. The diffusion-limited case (left) and mixed regime case (center) show expected average elevation values around 20°, while the reaction-limited case (right) shows higher than expected elevation values.

in both directions evolves from approximately 0.35-0.40 W/(m·K) to 0.15-0.20 W/(m·K). This insignificant difference in TT and IP effective thermal conductivity is likely caused by sample misalignment in the quartz tube; *i.e.*, the sample was not extracted with the quartz tube exactly perpendicular to the IP direction, resulting in a misalignment between the xy-plane of the sample and true IP direction of the material. As a result, in the reaction-limited case, the TT direction does not align with the z axis, and the IP direction does not align with the xy-plane, producing similar effective property values in both directions.

Alignment of each sample can be verified by examining the fiber elevation distribution of the first time point of each experiment measured in PuMA, as shown in Figure S8 (the full test can be seen in Figure 4). For both the diffusion-limited case (Figure S8, left) and the mixed regime case (Figure S8, center), fiber elevation distributions share a similar shape, with peak elevation at 0° and average fiber elevation near the expected 20° (see Methods section). In contrast, the reaction-limited case (Figure S8, right) shows a significantly higher peak in the distribution at 37° and a greater average elevation of 32°. Since elevation is the measured angle between the radial vector and its projection onto the xy-plane, this significant difference in fiber elevation for the reaction-limited case demonstrates misalignment between the xy-plane and the true IP direction of the material; with a significant tilt between the xy-plane and the IP direction, measured fiber elevation will increase, yielding an offset in the peak of the distribution and greater average values. While this misalignment in the reaction-limited case has no effect on the observations related to diffusion-reaction competition, it does explain the observation of similar IP and TT effective thermal conductivity values.

Movies

Supporting Movie S1. Time-resolved μ-CT visualization and in-depth slice-wise average porosity for tests 1–3, conducted at 100.5 kPa and 1320 K, 100.3 kPa and 1060 K, and 100.7 kPa and 945 K, respectively.

Supporting Movie S2. Time-resolved μ-CT visualization and in-depth slice-wise average porosity of tests 4-6, conducted at 28.5 kPa and 1375 K, 22.1 kPa and 1185 K, and 20.4 kPa and 1025 K, respectively.

Supporting Movie S3. Time-resolved μ-CT visualization and in-depth slice-wise average porosity of tests 7-9, conducted at 3.0 kPa and 1445 K, 2.4 kPa and 1220 K, and 2.4 kPa and 1055 K, respectively.

References

[1] Rosner, D. E. Scale effects and correlations in nonequilibrium convective heat transfer. *AIAA Journal* **1** (7), 1550–1555 (1963).

[2] Desai, P. N. & Qualls, G. D. Stardust entry reconstruction. *Journal of Spacecraft and Rockets* **47** (5), 736–740 (2010).

[3] Trumble, K. A., Cozmuta, I., Sepka, S., Jenniskens, P. & Winter, M. Postflight aerothermal analysis of the stardust sample return capsule. *Journal of Spacecraft and Rockets* **47** (5), 765–774 (2010).

[4] Kim, J. G. & Jo, S. M. Modification of chemical-kinetic parameters for 11-air species in re-entry flows. *International Journal of Heat and Mass Transfer* **169**, 120950 (2021).

[5] Panerai, F., Cochell, T., Martin, A. & White, J. D. Experimental measurements of the high-temperature oxidation of carbon fibers. *International Journal of Heat and Mass Transfer* **136**, 972–986 (2019).

[6] Badran, A. *et al.* Automated segmentation of computed tomography images of fiber-reinforced composites by deep learning. *Journal of Materials Science* **55** (34), 16273–16289 (2020).

[7] Ronneberger, O., Fischer, P. & Brox, T. Navab, N., Hornegger, J., Wells, W. M. & Frangi, A. F. (eds) *U-net: Convolutional networks for biomedical image segmentation.* (eds Navab, N., Hornegger, J., Wells, W. M. & Frangi, A. F.) *Medical Image Computing and Computer-Assisted Intervention – MICCAI 2015*, 234–241 (Springer International Publishing, Cham, 2015).

[8] Buschow, K. H. J. *et al.* (eds) *Encyclopedia of Materials: Science and Technology* (Elsevier, Oxford, 2001).

[9] Ferguson, J. C., Borner, A., Panerai, F., Close, S. & Mansour, N. N. Continuum to rarefied diffusive tortuosity factors in porous media from x-ray microtomography. *Computational Materials Science* **203**, 111030 (2022).

[10] Scoggins, J. B., Leroy, V., Bellas-Chatzigeorgis, G., Dias, B. & Magin, T. E. Mutation++: Multicomponent thermodynamic and transport properties for ionized gases in c++. *SoftwareX* **12** (2020).

[11] Vianna, R. S., Cunha, A. M., Azeredo, R. B. V., Leiderman, R. & Pereira, A. Computing effective permeability of porous media with fem and micro-ct: An educational approach. *Fluids* **5** (1), 16 (2020).

[12] Quintard, M. & Whitaker, S. Transport in ordered and disordered porous media V: Geometrical results for two-dimensional systems. *Transport in Porous Media* **15** (2), 183–196 (1994).

[13] Quintard, M. & Whitaker, S. Transport in ordered and disordered porous media: volume-averaged equations, closure problems, and comparison with experiment. *Chemical Engineering Science* **48** (14), 2537–2564 (1993).

[14] Whitaker, S. *The Method of Volume Averaging* (Springer Netherlands, 1999).

[15] Whitaker, S. Flow in porous media I: A theoretical derivation of Darcy's law. *Transport in Porous Media* **1** (1), 3–25 (1986).

[16] Whitaker, S. The Forchheimer equation: A theoretical development. *Transport in Porous Media* **25** (1), 27–61 (1996).

[17] Carbonell, R. G. & Whitaker, S. in *Heat and mass transfer in porous media* (eds Carbonell, R. G. & Whitaker, S.) *Fundamentals of Transport Phenomena in Porous Media* 121–198 (Springer, Davis, CA, 1984).

[18] Breugem, W.-P. *The influence of wall permeability on laminar and turbulent flows - Theory and simulations.* Ph.D. thesis, TU Delft (2004).

[19] Dias, B., Zibitsker, A. L., Meurisse, J. B. & Mansour, N. N. AIAA (ed.) *Towards a flow-material unified solver for heatshield modeling.* (ed.AIAA) *AIAA SCITECH 2023 Forum*, 2718 (American Institute of Aeronautics and Astronautics, National Harbor, MD, 2023).

[20] Gray, W. & Lee, P. On the theorems for local volume averaging of multiphase systems. *International Journal of Multiphase Flow* **3** (4), 333–340 (1977).

[21] Mansour, N. N., Panerai, F., Lachaud, J. & Magin, T. Flow mechanics in ablative thermal protection systems. *Annual Review of Fluid Mechanics* **56**, 549–575 (2024).

[22] Quintard, M. & Whitaker, S. Transport in ordered and disordered porous media II: Generalized volume averaging. *Transport in Porous Media* **14** (2), 179–206 (1994).

[23] Davit, Y. & Quintard, M. Technical Notes on Volume Averaging in Porous Media I: How to Choose a Spatial Averaging Operator for Periodic and Quasiperiodic Structures. *Transport in Porous Media* **119** (3), 555–584 (2017).

[24] Semeraro, F., Ferguson, J. C., Acin, M., Panerai, F. & Mansour, N. N. Anisotropic analysis of fibrous and woven materials part 2: Computation of effective conductivity. *Computational Materials Science* **186**, 109956 (2021).

[25] Lopes, P. C. *et al.* Simulation toolkit for digital material characterization of large image-based microstructures. *Computational Materials Science* **219**, 112021 (2023).

[26] Lopes, P. C. F., Semeraro, F., Pereira, A. M. B. & Leiderman, R. Enabling fem-based absolute permeability estimation in giga-voxel porous media with a single gpu. *Computer Methods in Applied Mechanics and Engineering* **434**, 117559 (2025).
